# Chemical Modifications in Aggregates of Recombinant Human Insulin Induced by Metal-Catalyzed Oxidation: Covalent Cross-Linking via Michael Addition to Tyrosine Oxidation Products

**DOI:** 10.1007/s11095-012-0755-z

**Published:** 2012-05-10

**Authors:** Riccardo Torosantucci, Olivier Mozziconacci, Victor Sharov, Christian Schöneich, Wim Jiskoot

**Affiliations:** 1 Division of Drug Delivery Technology Leiden/Amsterdam Center for Drug Research (LACDR), Leiden University, P.O. Box 9502, 2300 Leiden, The Netherlands; 2 Department of Pharmaceutical Chemistry, University of Kansas, 2095 Constant Avenue, Lawrence, KS 66047 USA

**Keywords:** human insulin, oxidation, Michael addition, aggregation, fragmentation

## Abstract

**Purpose:**

To elucidate the chemical modifications in covalent aggregates of recombinant human insulin induced by metal catalyzed oxidation (MCO).

**Methods:**

Insulin was exposed for 3 h at room temperature to the oxidative system copper(II)/ascorbate. Chemical derivatization with 4-(aminomethyl) benzenesulfonic acid (ABS) was performed to detect 3,4-dihydroxyphenylalanine (DOPA) formation. Electrospray ionization-mass spectrometry (ESI-MS) was employed to localize the amino acids targeted by oxidation and the cross-links involved in insulin aggregation. Oxidation at different pH and temperature was monitored with size exclusion chromatography (SEC) and ESI-MS analysis to further investigate the chemical mechanism(s), to estimate the aggregates content and to quantify DOPA in aggregated insulin.

**Results:**

The results implicate the formation of DOPA and 2-amino-3-(3,4-dioxocyclohexa-1,5-dien-1-yl) propanoic acid (DOCH), followed by Michael addition, as responsible for new cross-links resulting in covalent aggregation of insulin during MCO. Michael addition products were detected between DOCH at positions B16, B26, A14, and A19, and free amino groups of the N-terminal amino acids Phe B1 and Gly A1, and side chains of Lys B29, His B5 and His B10. Fragments originating from peptide bond hydrolysis were also detected.

**Conclusion:**

MCO of insulin leads to covalent aggregation through cross-linking via Michael addition to tyrosine oxidation products.

**Electronic supplementary material:**

The online version of this article (doi:10.1007/s11095-012-0755-z) contains supplementary material, which is available to authorized users.

## Introduction

During pharmaceutical production and storage, therapeutic proteins can be exposed to components that are able to induce metal-catalyzed oxidation (MCO) (1), i.e. redox active transition metals, peroxides and reductants (2,3). MCO of therapeutic proteins (4) such as recombinant human interferon alfa and recombinant human interferon beta has been reported to form highly immunogenic aggregates (5–7), providing evidence that protein oxidation can potentially lead to severe side reactions and loss of therapeutic effect. Furthermore, it has been shown that in diabetic complications (8), copper ion concentrations are higher than in normal subjects (9). Therefore, MCO is a potential cause of insulin degradation *in vivo* as well, when the production of reactive oxygen species (ROS) exceeds the endogenous antioxidant defense. Montes-Cortes *et al.* (10) showed that the carbonyl content of human insulin and the hydroxylation of phenylalanine, based on the formazan assay (11), is increased after exposure of insulin to the plasma of diabetic patients, which can contain high concentrations of oxidants (12). Moreover, in a recent paper we showed that MCO induces significant covalent aggregation of insulin *in vitro* (13). However, the underlying mechanisms of insulin aggregation through MCO are still unknown. Previous reports on the MCO of human insulin and glycated insulin indicate that both histidine residues are easily oxidized to 2-oxo histidine (2,9).

**Fig. 1:**
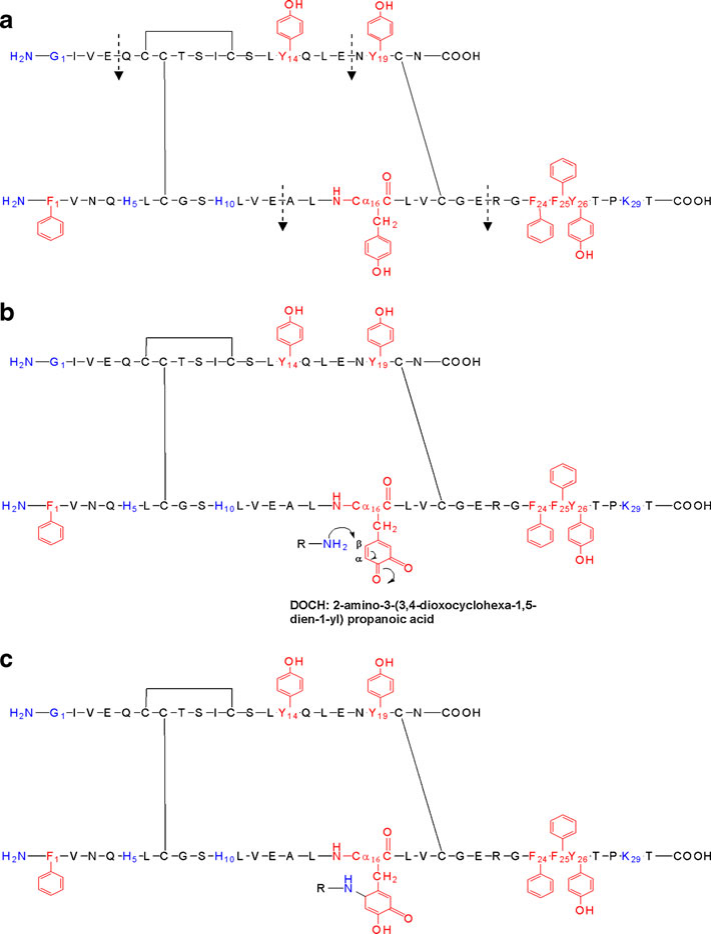
(**a**) primary structure of human insulin with the Glu-C endoproteinase digestion sites. In blue are shown the nucleophilic amino acids which can be involved in Michael addition. In red the amino acids that can be oxidized to DOPA and DOCH. (**b**) Michael addition on oxidized human insulin. The amino group that initiates a nucleophilic reaction with the α,β unsaturated carbonyl compound (DOCH), represents one of the nucleophilic amino acids of human insulin (in blue). (**c**) Product of Michael addition on oxidized insulin.

**Scheme 1:**
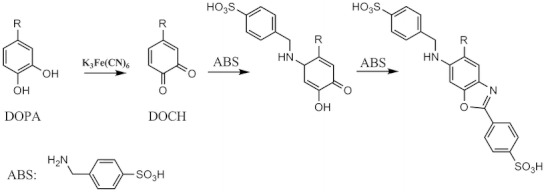
Chemical derivatization of DOPA and DOCH with ABS.

Here we provide a more detailed analysis of the chemical modifications that occur during the MCO of recombinant human insulin, induced by Cu^2+^ and L-ascorbic acid as a representative electron donor (14–16), focusing on the amino acids that are modified and on the chemical mechanisms that lead to cross-linked insulin, as a consequence of the modifications in the primary structure. Since previous studies have demonstrated the presence of DOPA in proteins like cataractous lens proteins (17) and in atherosclerotic lesions (18), emphasis was placed on the detection of oxidation products arising from the four Tyr and the three Phe contained in insulin and, especially, on the formation of 2-amino-3-(3,4-dioxocyclohexa-1,5-dien-1-yl) propanoic acid (DOCH), which constitutes a Michael acceptor for nucleophilic addition. Figure 1, panel a, displays the primary structure of insulin, the Glu-C digestion sites, the amino acids (in blue) with nucleophilic function, and the amino acids (in red) that can be oxidized to 3,4-dihydroxyphenylalanine (DOPA) and DOCH; Fig. 1b representatively illustrates the Michael addition on DOCH at position B16, and Fig. 1c shows the product of Michael addition on oxidized insulin. Complementary detection of DOPA and DOCH was achieved using mass spectrometry and fluorogenic tagging with 4 (aminomethyl)benzene sulfonic acid (ABS) (Scheme 1), according to the method reported recently by Sharov *et al.* (19,20) Our results indicate that all aromatic amino acids of insulin, i.e. His, Tyr and Phe, are subject to MCO, predominantly yielding mono- and/or dihydroxylation products (or their respective oxidation products, i.e. DOCH from DOPA). Important for the mechanistic analysis of covalent protein aggregation, we detected cross-links between DOCH at positions B16, B26, A14, and A19 and free amino groups of the N-terminal amino acids Phe B1 and Gly A1, and the side chains of Lys B29, His B5 and His B10.

## Materials and Methods

### Materials

Recombinant human insulin containing 0.4 % (w/w) zinc was provided by Merck (Oss, The Netherlands). L-ascorbic acid, ethylenediaminetetraacetic acid sodium salt (EDTA), copper dichloride, arginine, monobasic and dibasic sodium hydrogen phosphate, ammonium bicarbonate (ABI), dithiothreitol (DTT), iodoacetamide (IAM) and the solvents glacial acetic acid and acetonitrile were purchased from Sigma–Aldrich (St.Louis, MO, USA). Millipore Q water was used for the preparation of all the formulations and solutions. All chemicals were of analytical grade and used without further purification. ABS was synthesized according to a published procedure (21). Glu-C endoproteinase used in this study was purchased from Promega (Madison, WI, USA). Centrifugal filter units with a volume capacity of 4 mL and a molecular weight cut-off of 3 kDa were purchased from Millipore (Billerica, MA, USA). Cassette dialysis slides with a 2 kDa cut-off were purchased from Thermo Scientific (Asheville, NC, USA).

### Metal-Catalyzed Oxidation of Insulin

Insulin oxidation was performed at three pH values: pH 7.4 in 50 mM sodium phosphate buffer (PB), pH 3.0 in 50 mM sodium citrate buffer (CB) and at pH 8.0 in 250 mM ammonium bicarbonate (ABI). For all the oxidation reactions, insulin was first dissolved in 0.1 M hydrochloric acid and then diluted into the corresponding buffer. Depending on the desired pH, 0.1 M sodium hydroxide was used. When CB was used, the final pH of 3 was achieved without further addition of base. Insulin concentration was measured by UV spectroscopy using a molecular weight of 5.8 kDa and an extinction coefficient of 6200 M^−1^ cm^−1^ at 276 nm (22). Further dilutions in PB, ABI or CB were performed to obtain a final insulin concentration of 1 mg/mL. Controls included 1 mg/mL of insulin in 50 mM PB, pH 7.4; in 50 mM CB, pH 3.0; and in 250 mM ABI, pH 8.0. MCO was performed by addition to 1 mL of 1 mg/mL insulin, 100 μL of 0.4 mM CuCl_2_ in 50 mM PB, pH 7.4, or in 50 mM CB, pH 3, or in 250 mM ABI, pH 8, depending on the desired pH, to a final concentration of 40 μM CuCl_2_. The reaction was performed in 2-mL Eppendorf tubes covered with aluminum foil to protect the reaction mixture from light. After 10 min of incubation of insulin with Cu^2+^, to allow copper to bind to insulin, the oxidation reaction was started by the addition of 110 μL of 40 mM L-ascorbic acid in 50 mM PB, pH 7.4, or in 50 mM CB, pH 3, or in 250 mM ABI, pH 8, depending on the desired pH, to a final concentration of 4 mM. The reaction was quenched after 3 h of incubation at room temperature by adding 12.1 μL of a 100 mM EDTA in 50 mM PB, pH 7.4, or in 50 mM CB, pH 3, or in 250 mM ABI, pH 8, depending on the desired pH, to a final concentration of 1 mM (15). To monitor the presence of protein fragments, the oxidation was also performed (in PB with the same amount of copper and L-ascorbic acid) at room temperature and at 37°C for 24 h before quenching. The oxidized samples were extensively dialyzed at +4°C against 50 mM ammonium bicarbonate (ABI) buffer, pH 8.0, for 24 h. In another experiment, the oxidized sample in 50 mM sodium citrate buffer pH 3, was extensively dialyzed at + 4°C in 50 mM sodium phosphate buffer, pH 7.4, using centrifugal filter units. After that the sample was left to equilibrate for 12 h at room temperature.

### ABS-Derivatization of Undigested Samples

Solutions of native insulin and insulin oxidized at pH 3.0 (in CB) and pH 7.4 (in PB), dialyzed into 50 mM ABI, were treated with 0.1 M sodium hydroxide to a final pH of 9.0. Subsequently, to 100 μL of these solutions, 11 μL of a solution of 100 mM ABS dissolved in water were added to a final concentration of 10 mM. Next, 1.1 μL of 50 mM K_3_Fe(CN)_6_ dissolved in water was added to a final concentration of 0.5 mM. The reaction was conducted for 1 h at room temperature before performing SEC analysis. Controls for non-specific fluorescence included derivatization reagents (without protein) incubated under the same conditions and non-derivatized protein.

### Reduction, Alkylation and ABS-Derivatization of Insulin

Solutions of native insulin and oxidized insulin, dialyzed into 50 mM ABI, pH 8.0, were reduced using 50 mM dithiothreitol (DTT), freshly prepared in 50 mM ABI, pH 8.0, added to a final concentration of 5 mM. The samples were incubated for 45 min at 45°C using a thermo heating bath (Thermo Scientific, Asheville, NC, USA). Subsequently, 200 mM iodoacetamide (IAM), freshly prepared in 50 mM ABI, pH 8.0, was added to a final concentration of 20 mM. The pH of 100 μL of dialyzed, reduced and alkylated insulin samples was adjusted to 9.0 with 0.1 M sodium hydroxide. Then, 11 μL of a solution of 100 mM ABS dissolved in water were added to a final concentration of 10 mM. Subsequently, 1.11 μL of 50 mM K_3_Fe(CN)_6_ dissolved in water was added to a final concentration of 0.5 mM. The reaction was conducted for 1 h at room temperature before performing digestion with Glu-C.

### Glu-C Digestion

The proteolytic digestion was performed after reduction, alkylation and ABS-derivatization of insulin, by incubating the samples with Glu-C endoproteinase in a ratio insulin: Glu-C endoproteinase 10:1 (w/w), overnight at 37°C.

### Size-Exclusion Chromatography

An Insulin HMWP column (7.8 × 300 mm, Waters, Milford, MA, USA) was connected to a Shimadzu HPLC system (UFLC Shimadzu Instrument equipped with two LC-20AT pumps, Columbia, MD) coupled to a photo-diode array detector (Shimadzu, SPD-M20A) and a fluorescence detector (Shimadzu, RF-20A). The photo diode array detector allowed for the recording of the UV spectrum in the region between 200–800 nm. Undigested insulin samples, without ABS treatment, were injected to calculate the percentages of the aggregates and to measure tyrosine fluorescence. Moreover, undigested insulin samples that were treated with ABS were injected to measure the benzoxazole fluorescence, which can be formed only in presence of DOPA and/or DOCH. The percentage of aggregates was calculated based on peak areas of the UV peaks at 276 nm (chromatograms not shown), as reported by Hermeling *et al.* (5,6). The fluorescence detector was set at various different excitation (Ex) and emission (Em) wavelengths, depending on the type of analysis: Ex 275 nm/Em 302 nm was used for monitoring Tyr fluorescence and Ex 360/Em 490 nm was used for the detection of the benzoxazole fluorescence of the ABS-derivatized samples. The mobile phase was composed of a mixture of 1 g/L L-arginine in water/acetonitrile/glacial acetic acid 65:20:15 (v/v/v) as reported in the United States and European pharmacopeias (23,24). The elution buffer was freshly prepared, filtered using a regenerated cellulose filter (Sartorius Stedim Biotech, Arvada, CO) and degassed prior to use.

### Steady-State Fluorescence Spectroscopy

The benzoxazole fluorescence of the ABS-derivatized samples was measured upon excitation at 360 nm. The emission spectra were recorded from 490 nm to 600 nm with a 5-nm bandwidth on a Shimadzu RF-5000U spectrofluorometer. To monitor the presence of dityrosine, the non-derivatized, oxidized samples were analyzed after dialysis in 50 mM ABI, pH 8.0, using an excitation wavelength of 315 nm and detection at 420 nm (25).

After ABS-derivatization and dialysis, sample volumes were diluted with 50 mM ABI to 0.5 mL. Spectra were recorded in 0.5 mL 1-cm light-pass fluorescence cuvettes (Hellma, Plainview, NY, USA). Controls for non-specific fluorescence included samples without a substrate or reagents incubated under the same conditions.

### Mass Spectrometry

Digested and non digested peptides were analyzed by means of an LTQ-FT hybrid linear quadrupole ion trap Fourier transform ion cyclotron resonance (FT-ICR) mass spectrometer (Thermo-Finnigan, Bremen, Germany) (26) and a SYNAPT-G2 (Waters Corporation, Milford, MA), both located in the Mass Spectrometry Laboratory of the University of Kansas, under the conditions as described by Ikehata *et al.* (26). In short, peptides were separated on a reversed-phase LC Packings PepMap C18 column (0.300 × 150 mm) at a flow rate 10 μL/min with a linear gradient from 0 to 65 % acetonitrile in 0.06 % aqueous formic acid over a period of 55 min using LC Packing Ultimate Chromatograph (Dionex). LC-MS experiments were performed in a data-dependent acquisition manner using Xcalibur 2.0 software (Thermo Scientific). Five most intensive precursor ions in a survey MS1 mass spectrum acquired over a mass range of 300–2000 m/z were selected and fragmented in the linear ion trap by collision-induced dissociation. The ion selection threshold was 500 counts. The MS/MS spectra obtained were analyzed with the software MassMatrix (27–30). MassMatrix was used to generate the theoretical fragment tables of the b and y ions of the different oxidized and cross-linked products. The theoretical fragments were compared to the experimental MS/MS spectra to validate the structures, which were taken into consideration only if the difference between the theoretical and the experimental m/z of the parent ion (and the fragment ions) was strictly below 0.1 Da. The SYNAPT-G2 instrument was operated for maximum resolution with all lenses optimized on the [M + 2 H]^2+^ ion from the [Glu]^1^-fibrinopeptide B. The cone voltage was 30 V and Ar was admitted to the collision cell. The spectra were acquired using a mass range of 50–2000 m/z. The data were accumulated for 0.7 s per cycle. The CID data, at the MS^2^ level, acquired with the FT-ICR instrument were obtained after an attenuation of the parent ion of 35 %. The mass window to collect the parent ion was fixed to 0.1 Da. Deconvolution of the electrospray ionization data of the undigested insulin was obtained using the maximum entropy distribution algorithm implemented in the Masslynx MaxEnt software (Waters Corporation, Milford, MA) using an adduct of 1 proton. Assuming a normal statistical distribution of the noise, a uniform Gaussian with a width at half height of 0.5 Da was used. A number of fifty iterations and a range between 0–36000 Da were used to build the most probable mass spectrum of the parent ions.

## Results

To guide the reader through the results, these will be presented in three sections: 1) Aggregation Profile of Oxidized Insulin and Elucidation of Aggregation Mechanism, 2) MS Analysis of Undigested Insulin Samples, and 3) Identification of Chemical Modifications by MS/MS Analysis of Reduced, Alkylated, ABS-Derivatized and Digested Samples.

### Aggregation Profile of Oxidized Insulin and Elucidation of Aggregation Mechanism

**Fig. 2:**
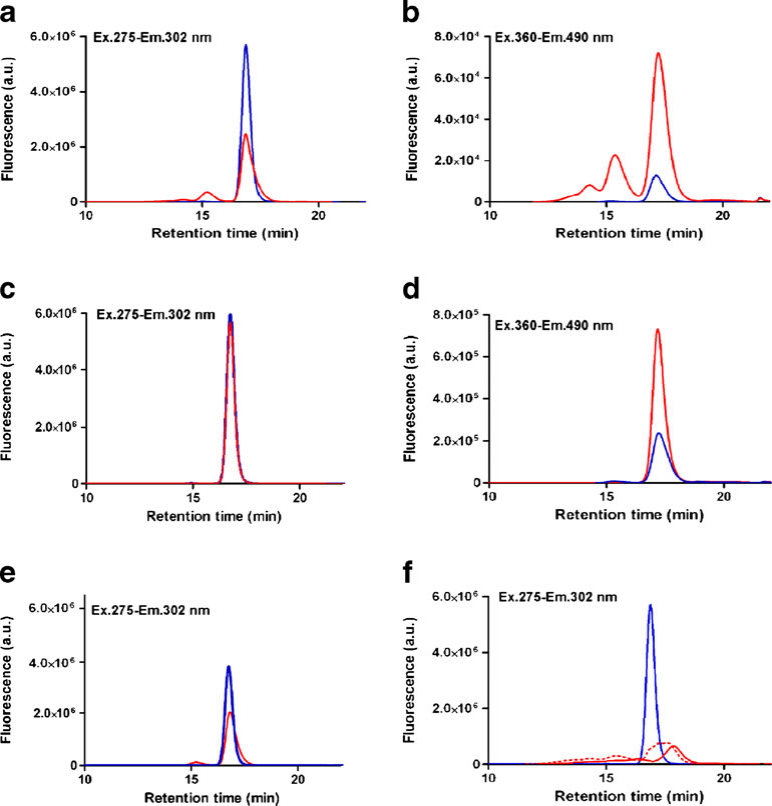
SEC with intrinsic tyrosine fluorescence detection (panels **A**, **C**, **E**, **F**; Ex 275 nm-Em 302 nm) and ABS fluorescence detection (panels **B**, **D**): (**A**) insulin oxidized in PB 50 mM, pH 7.4, for 3 h at room temperature (in red) and its control, untreated insulin (in blue); (**B**) oxidized insulin (pH 7.4), derivatized with ABS (in red) and its control, untreated insulin derivatized with ABS (in blue); (**C**) insulin oxidized in 50 mM sodium citrate, pH 3.0, for 3 h at room temperature (in red) and its control, untreated insulin (in blue); (**D**) oxidized insulin (pH 3.0), derivatized with ABS (in red) and its control, untreated insulin derivatized with ABS (in blue); (**E**) insulin oxidized in 50 mM sodium citrate, pH 3.0, for 3 h at room temperature, exchanged in 50 mM PB, pH 7.4, for 12 h at room temperature (in red) and its control, oxidized insulin (pH 3) (in blue); (**F**) insulin oxidized in 50 mM PB, pH 7.4, for 24 h, at room temperature (dotted red line) and at 37°C (solid red line), untreated insulin (blue line).

Investigation of the chemical mechanisms, as well as the aggregation profile, was achieved with SEC. This technique was employed for several purposes: to detect and quantify the percentage of monomer loss, and the formation of covalent dimers and high-molecular-weight oligomers (HMWO) during MCO, by using UV detection at 276 nm (note that the composition of the mobile phase does not allow us to estimate the amount of non-covalent aggregates since these will be dissociated because of the organic solvent and the low pH of the eluent (31)); to monitor Tyr fluorescence in oxidized insulin, by using an excitation wavelength of 275 nm with emission set at 302 nm, and to detect the development of DOPA and DOCH through derivatization with ABS. Furthermore, we used SEC to monitor the presence of fragments after prolonged oxidation. The percentages found (Table I) are in agreement with our previous results (13). MCO under our applied standard conditions leads to a loss of 25.6 % of monomer and the formation of 18.8 % of dimer and 6.8 % of HMWO (Fig. 2a). The derivatization with ABS, followed by SEC analysis, allowed the detection of DOPA and DOCH, in the monomer, as well as in the aggregates (Fig. 2b). The overall results point to the presence of Tyr and Phe oxidation products in all insulin species formed during MCO. A much weaker overall fluorescent signal was detected for the derivatized non-oxidized insulin (Fig. 2b), suggesting that a low percentage of DOPA was already present in the starting material, which could have been oxidized during production, storage and handling. The fluorescence spectra of the ABS derivatized samples (data not shown) were in agreement with the fluorescence detection performed with SEC: a low but measurable fluorescence emission was found for the native insulin derivatized with ABS and a 6-fold higher fluorescence signal was obtained for the oxidized insulin derivatized with ABS. Aggregated insulin was almost completely absent when MCO was performed at pH 3.0 (Fig. 2c). This result suggests that the aggregation process, which appears to be mediated by nucleophilic reactions with DOCH, is inhibited at lower pH, likely due to protonation of free amino groups and His residues. Nevertheless, DOPA and DOCH were detected after derivatization with ABS (Fig. 2d), indicating that the lower pH did not inhibit insulin oxidation. Hence, it may be possible that insulin oxidized at pH 3.0 would still aggregate if the pH is increased after oxidation. To test this hypothesis, the buffer of the insulin sample oxidized at pH 3.0 was subsequently exchanged with 50 mM sodium phosphate buffer, pH 7.4, using centrifugal filter units. The sample was left to equilibrate for 12 h at room temperature before SEC analysis. Figure 2e and Table I represent the chromatograms and the percentages of aggregates for this experiment, displaying an increased amount of insulin dimer and trimer after the buffer change. Altogether these results indicate that Michael addition, the addition of a nucleophile to an α,β-unsaturated carbonyl compound (32), may be the main mechanism of covalent insulin aggregation during MCO. Moreover, this experiment disproves a major role of S-S scrambling in insulin aggregation during MCO. However, the occurrence of non-covalent (besides covalent) aggregation cannot be fully excluded.

**Table I:** Percentages of Monomer, Dimer and High-Molecular-Weight Oligomers (HMWO) for the Insulin Oxidized Under Different Conditions and Native Insulin

Sample	%Monomer	%Dimer	%HMWO
Oxidized insulin in PB, pH 7.4	74.4 ± 0.9	18.8 ± 1.4	6.8 ± 1.2
Oxidized insulin in ABI, pH 8.0	96.1 ± 0.7	3.9 ± 0.7	ND^*a*^
Oxidized insulin in sodium citrate, pH 3	99.2 ± 0.3	0.8 ± 0.3	ND
Oxidized insulin in sodium citrate, pH 3.0, spiked into PB, pH 7.4	88.8 ± 1.3	9.3 ± 1.1	1.9 ± 0.1
Native insulin in PB, pH 7.4	99.4 ± 0.2	0.6 ± 0.2	ND

Based on SEC analysis with UV detection at 276 nm; data represent mean ± standard deviation of 3 individual batches

^*a*^ND = not detected

**Fig. 3:**
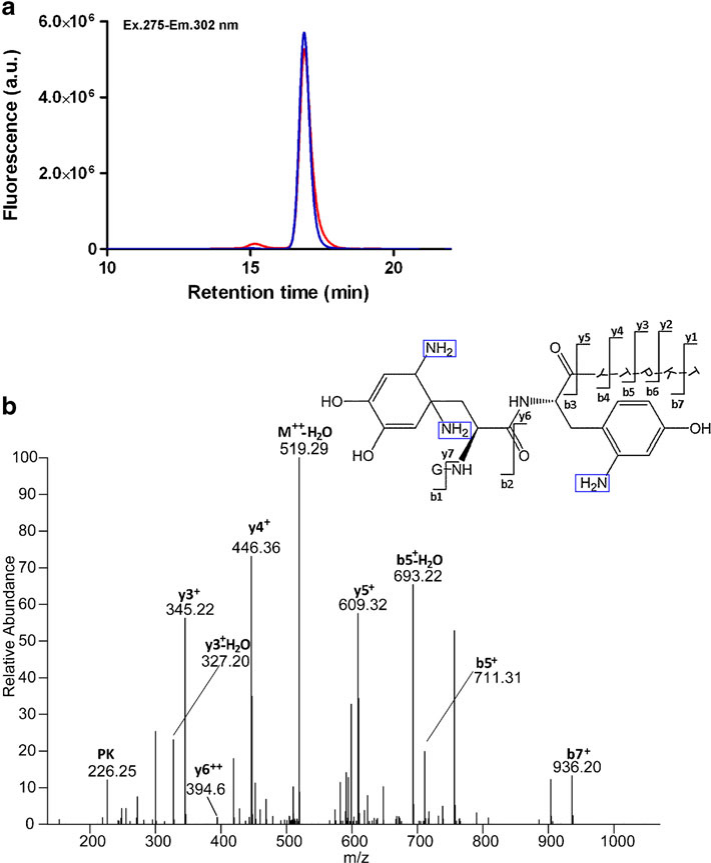
(**a**) SEC with intrinsic tyrosine fluorescence detection (Ex 275 nm-Em 302 nm) of insulin oxidized for 3 h at room temperature in ABI 0.25 M (in red) and its control (in blue). (**b**) MS/MS of peptidic fragment obtained from oxidized insulin in ABI 0.25 M, reduced (DTT), alkylated (IAM), and digested with Glu-C. The amino groups added through Michael addition to the oxidized phenylalanine are highlighted in blue.

Additional evidence for Michael addition was provided in an experiment in which we performed the oxidation of insulin at pH 8.0, in the presence of a relatively high concentration (0.25 M) of ammonium bicarbonate. Under these conditions ammonia competes with nucleophilic functional groups of insulin (i.e. the N-terminus of Gly A1 and Phe B1, and the side chains of His B5, B10 and Lys B29) for the addition to DOCH, thereby inhibiting aggregates formation, as shown in Fig. 3a and indicated by the low percentages of covalent aggregates measured (Table I). This was confirmed with FT-ICR MS analysis, performed on oxidized insulin in ABI, after reduction, alkylation and Glu-C digestion. As can be seen in the chemical structure representatively depicted in Fig. 3b, two amino groups (highlighted in blue) are covalently bound to the first Phe residue and one amino group is covalently bound to the second Phe residue, indicating that Michael addition occurred between both oxidized Phe residues and ammonia originating from the buffer. These Michael addition products are generated by addition of the nucleophiles to the two β-type carbon atoms in the oxidation product DOCH, and are consistent with the mass increases of the respective original Phe residues; however, due to the low abundance of sample we did not confirm the regiochemistry of nucleophile addition by NMR. The most relevant ions in the MS/MS spectra, which allow to confirm the addition of ammonia to DOCH, are b5^+^, y6^++^ and y5^+^. The first, with m/z 711.31 of a singly charged ion, shows the sequence G_B23_FFYT_B27_ plus the additional molecular weight of three oxygen atoms and three ammonia molecules. These data suggest that the sequence PKT is neither oxidized, nor derivatized with amino groups. The doubly charged ion y6^++^, with m/z 394.6, related to the sequence F_B25_YTPKT_B30_, shows that one oxygen and one ammonia molecule are incorporated into this sequence. Of course the Tyr residue could be the target for this incorporation; however, the ion y5^+^ argues against this hypothesis where the singly charged ion with m/z 609.32 represents the unmodified sequence Y_B26_TPKT_B30_.

To evaluate the potential of MCO to induce fragmentation, already reported by other authors for the MCO of glycated insulin (9,33), and by our group for the MCO of PEGylated insulin (13), human insulin was oxidized in 50 mM PB, pH 7.4, for 24 h at room temperature and 37°C. Figure 2f clearly shows the elution of species after the monomer peak, which indicates the formation of lower molecular weight species.

### MS Analysis of Undigested Insulin Samples

The FT-ICR MS analysis presented in this section was performed on oxidized insulin and native insulin (our control), prior to reduction, alkylation, ABS-derivatization and digestion with Glu-C. This analysis was executed to monitor the formation of oxidized monomers and to confirm the MCO-induced fragmentation, as well as to quantify these species relative to native insulin.

#### Oxidation

**Fig. 4:**
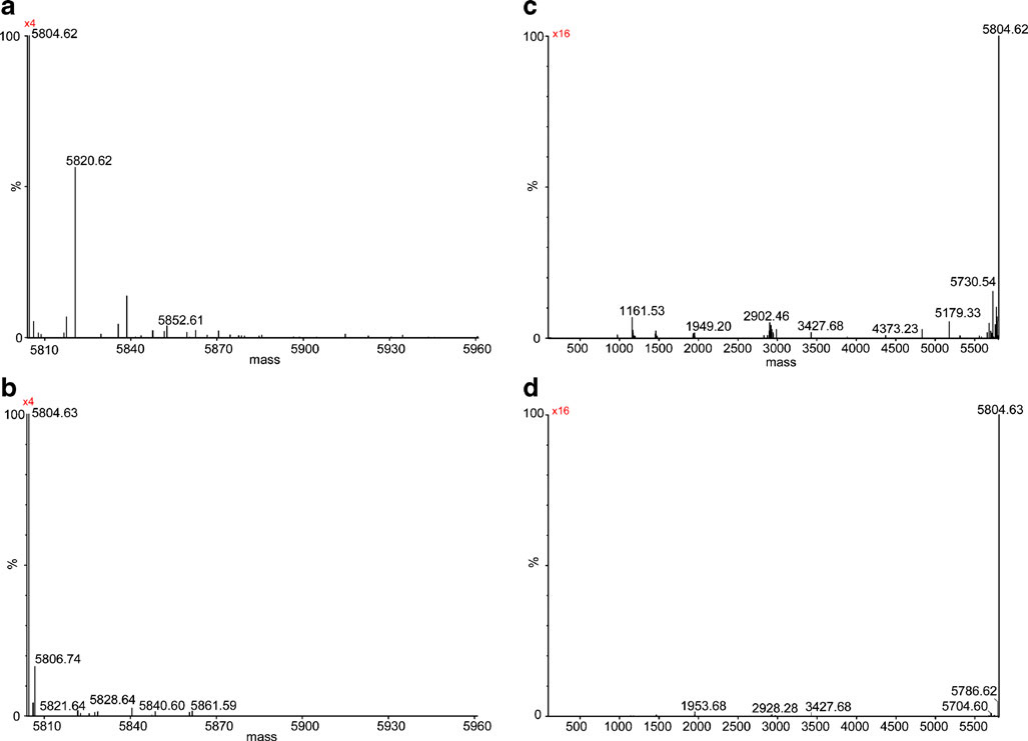
Mass spectrum (MS) of undigested insulin and undigested insulin oxidized for 3 h at room temperature in 50 mM PB, pH 7.4. Spectra are shown for oxidized insulin (**a**, **c**) and native insulin (**b**, **d**) in the mass range 5805–5961 to show the oxidized monomer (**a**, **b**) and in the mass range 100–5805 to show the fragments (**c**, **d**). Spectra were obtained using the maximum entropy distribution algorithm implemented in the Masslynx MaxEnt software (Waters Corporation, Milford, MA) using an adduct of 1 proton. (Please note that 5804.63 represents the molecular mass of unprotonated insulin.)

**Fig. 5:**
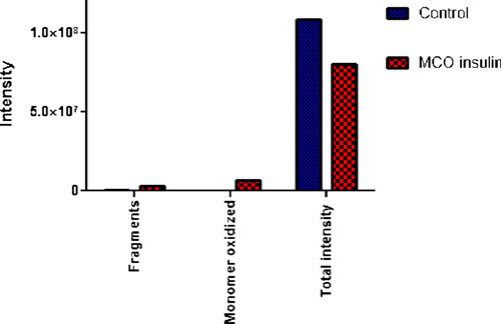
(**A**) Plot representing the intensity of fragments and oxidized monomer relative to the total intensity of all detected peaks for native insulin (control, in blue) and insulin oxidized for 3 h at room temperature in 50 mM PB, pH 7.4 (in red). Data are derived from the spectra reported in Fig. 4.

Human insulin contains three Phe and four Tyr residues. Oxidation of Phe to DOPA would require the incorporation of two oxygen atoms. Instead, oxidation of Tyr to DOPA would be represented by the incorporation of one oxygen atom. Hence, it should be possible to detect mass increases corresponding to ten atoms of oxygen per monomer, considering DOPA as oxidation product of Phe and Tyr. Based on this number, after deconvoluting each MS spectrum by using the maximum entropy distribution algorithm (Fig. 4), we added up the intensities observed for all monomers containing additional oxygen atoms (n = 0-10) (i.e., for the masses 5,804.63 + 16, 5804.63 + 32 + … + 5,804.63 + 160 Da). The resulting intensity of oxidized monomer in insulin exposed to MCO was found to be ~300 fold higher than that in non-oxidized insulin (Fig. 5), suggesting that the amount of insulin monomer containing oxygen had increased dramatically after MCO. These calculations are possible because after deconvolution of the mass-to-charge ratios, the absolute intensity of each ion is conserved. For better clarity, the oxidized monomer in insulin exposed to MCO and that of the non-oxidized insulin are presented in the mass range 5,805-5,961 (Fig. 4a, b). Figure 4a shows that the intensity of the mass signal at 5820.62 Da (indicating incorporation of one oxygen atom) and the intensity of the mass signal at 5852.61 Da (indicating incorporation of three oxygen atoms) are much more intense after oxidation. Note that signals indicating incorporation of several other numbers of oxygen atoms were detected too, although with lower intensity. Although MS analysis cannot detail which amino acid is exactly oxidized, our calculation provides a first estimation for the extent of MCO in the insulin monomer.

#### Fragmentation

In order to detect and quantify the yield of insulin fragments relative to native insulin and to understand if fragmentation, measured by SEC, occurred during sample preparation for analysis (reduction, alkylation, ABS-derivatization and digestion) or prior to sample preparation, i.e. during the exposure of insulin to Cu^2+^ and L-ascorbate, MS analysis of native and oxidized insulin was performed prior to and after reduction, alkylation, ABS-derivatization and digestion. Figure 4c and d represent a zoom (16X) into the mass region between 100 and 5805 Da: clearly the oxidized insulin contains new peptides of a lower molecular weight compared to insulin. We processed the MS spectra of oxidized and non-oxidized insulin as follows: 1) deconvolution using the maximum entropy distribution algorithm; 2) sum of the intensities of all the fragments with a molecular weight between that of glycine (the smallest amino acid in insulin) and the molecular weight measured for insulin (i.e. 5804.63 Da) minus the molecular weight of glycine, after hydrolysis. The procedure that was employed is summarized in formula 1:

1 $$ {I_{total}} = \mathop \sum \limits_{i = j}^k {\left[ {Fragment} \right]_i} \left\{ {\matrix{{*{20}{c}} {j = Mw\left( {Gly} \right) = 75.03} \\ {k = Mw(Insulin - Gly + {H_2}O)} \\ {k = 5804.63 - 75.03 + 18} \\ {\left[ {Fragment} \right]i = Mw of the {i^{th}}fragment} \\ } } \right. $$

The MS spectra of the oxidized and native insulin that were used for this calculation are displayed in Fig. 4c and d, respectively. Figure 5 represents the histograms plotted using the intensity values of the same spectra. The total intensity of the control (i.e. native insulin), was calculated between masses of 0 Da and 36,000 Da, i.e. until just beyond the theoretical molecular weight of the insulin hexamer. Based on this calculation, the total yield of fragments in the non-oxidized control was 0.3 % compared to 3.1 % in insulin exposed to Cu^2+^ and L-ascorbate (percentages have been calculated dividing the total intensity of the fragments over the total intensity of the native insulin used as a control). For a better view of the fragments, we provide a zoom of the range between 100–5,805 Da of native insulin and oxidized insulin (Fig. 4c, d). Altogether these results suggest that MCO induces peptidic fragmentation. We did not explore in more detail the mechanism(s) by which such fragmentation occurs, nor did we search for all the possible cross-link in which the new N-termini could be involved. However, we did note the generation of new cross-links between insulin and such fragments (see below), which contain new N-termini and C-terminal amino acids not generated by Glu-C digestion.

### Identification of Chemical Modifications by MS/MS Analysis of Reduced, Alkylated, ABS-Derivatized and Digested Samples

The FT-ICR MS analysis of oxidized and native insulin (control) was performed after reduction, alkylation, ABS-derivatization and digestion with Glu-C, by injecting the samples into the mass spectrometer by liquid chromatography. The aim of this analysis was to map the chemical modifications in the primary structure of insulin, to identify the nonreducible cross-links involved in insulin aggregation and to identify the fragments. Because different kind of oxidized, ABS-derivatized, and non-oxidized fragments were found, we hereafter define “fragments” as peptides resulting from at least one cleavage site that differs from the one expected from proteolytic digestion by Glu-C (i.e. after glutamic acid; Glu; E) and we define “Glu-C fragments” as peptides originating from the expected proteolytic cleavage sites of Glu-C. Table II lists all the detected “Glu-C fragments” (oxidized and ABS-derivatized) and “fragments” (oxidized, ABS-derivatized and non-oxidized). Non-oxidized Glu-C fragments were measured for both oxidized and native insulin but are not listed in Table II, since their presence was expected and represents the majority of Glu-C fragments. MS/MS spectra of non-oxidized fragments (available in the supplementary material) are only briefly discussed in this manuscript, because the main focus of our work was to elucidate the chemical mechanisms of insulin aggregation during MCO. As displayed in Table II, the following section is divided into the following subsections: Oxidized Glu-C Fragments, ABS-Derivatized Glu-C Fragments, Oxidized Fragments, ABS-Derivatized Fragments, Non-oxidized Fragments. Regarding the description of these fragments, the prefixes and subscripts A and B refer to sequences from the A and B chain, respectively, while b and y refer to the cleavage sites according to the nomenclature introduced by Roepstorff (34).

**Table II:** Fragments Identified in Reduced, Alkylated, ABS-Derivatized and Digested (Glu-C) Insulin and Oxidized Insulin, as Determined by Mass Spectrometry

Samples	Glu-C fragments oxidized	Glu-C fragments ABS-derivatized	Fragments oxidized	Fragments ABS-derivatized	Fragments Non-oxidized
Oxidized insulin	R_B22_GFF^*a*^Y^*a*^TPKT_B30_ Fig. S1 A, B, C	R_B22_GFFY^*c*^TPKT_B30_ Fig. S5	F^*a*^_B1_VNQHLCG_B8_ Fig. S7	A_B14_LY^*c*^LVC_B19_Fig. S10	L_A13_YQLE_A17_ Fig. S13
	A _B14_LY^*a*^LVCGE_B21_ Fig. S2	R_B22_GFFY^*d*^TPKT_B30_ Fig. S6	E_B21_RGF^*b*^FYTPKT_B30_ Fig. S8	S_A12_LY^*c*^QLE_A17_ Fig. S11	I_A10_CSLY_A14_ Fig. S14
	F^*a*^_B1_VNQHLCGSHLVE_B13_ Fig. S3		F^*a*^_B1_VNQHLCGSHLVEAL_B15_ Fig. S9	S_A12_LY^*d*^QLE_A17_ Fig. S12	S_A12_LYQLE_A17_ Fig. S15
	F^*b*^_B1_VNQHLCGSHLVEALYLVCGE_B21_ Fig. S4		H_B5_LCGSH^*a*^LVEAL_B15_ MS/MS not shown		S_A12_LYQL_A16_ Fig. S16
			F_B1_VNQH^*a*^LCGS_B9_ MS/MS not shown		Y_A14_QLE_A17_ Fig. S17
			F_B1_VNQH^*a*^LCGSHL_B11_ MS/MS not shown		G_B8_SHLVE_B13_ Fig. S18
					H_B10_LVEAL_B15_ Fig. S19
					F_B1_VNQHLCGS_B9_ Fig. S20
					Y_B16_LVC_B19_ Fig. S21
					G_B20_ERGFFY_B26_ Fig. S22
					G_A1_IVEQC_A6_ Fig. S23
					A_B14_LYLVCG_B20_ Fig. S24
					Q_A15_LENYCN_A21_ Fig. S25
					L_B6_CGSHLVE_B13_ Fig. S26
Insulin	R_B22_GFF^*a*^Y^*a*^TPKT_B30_ Fig. S1 A, B, C				G_B23_FFYTPKT_B30_ Fig. S27
					L_A13_YQLE_A17_ Fig. S13

oxidation of insulin was performed by MCO for 3 h at room temperature in 50 mM PB, pH 7.4

^*a*^incorporation of one oxygen atom

^*b*^incorporation of two oxygen atoms

^*c*^incorporation of one ABS molecule

^*d*^incorporation of two ABS molecules

#### Oxidized Glu-C Fragments

##### R_B22_GFF*Y*TPKT_B30_

The C-terminal region of chain B contains two Phe and one Tyr in positions B24, B25 and B26, respectively. DOPA and DOCH can arise from the oxidation of all these residues. Thus it becomes of primary importance to identify the ions, which allows us to discriminate between different oxidation products. The MS/MS spectra reported in supplementary Fig. S1A provides evidence for the oxidation of Phe B25 to Tyr where * indicates the incorporation of one oxygen atom. The ion b3^+^, despite its low intensity, indicates that Phe B24 is present in the native, non-oxidized state, where m/z 361.2 corresponds to the singly charged ion of the sequence RGF. Instead, the ion b4^+^ provides evidence for oxidation of Phe B25. A careful analysis of the spectra (Figures S1B) reveals the contemporary presence of two different y5^+^ ions, indicated as (*F4*)y5^+^ with m/z 625.5 (which corresponds to a structure containing Tyr B26 oxidized) and, (*HO-F4*)y5^+^ with m/z 609.4 (which corresponds to a structure containing Phe B25 oxidized). Panel C shows the b4^+^ ions for these two coexisting structures, again representing oxidation of Phe B25 or Tyr B26, respectively. Hence, both Phe B25 and Tyr B26 are targets for the incorporation of one oxygen atom.

##### A_B14_LY*LVCGE_B21_

Supplementary Figure S2 displays the MS/MS data for the peptide ALY*LVCGE, which contains an expected Glu-C cleavage site, where Y* represents the incorporation of one oxygen into Tyr, i.e. the formation of DOPA (the Cys residue is alkylated with IAM). This product is confirmed through the presence of the ions b3^+^-b5^+^ (although b3^+^ and b4^+^ show low intensities) and y5^+^. The singly charged ions b3^+^, b4^+^ and b5^+^ indicate that the oxygen is in one of the following sequences: ALY, ALYL, or ALYLV. The singly charged ion y5^+^ indicates that the sequence LVCGE is not oxidized. Therefore, oxygen incorporation must have occurred on one of the first three amino acids, ALY, where Tyr represents the most oxidation-sensitive target amino acid. Further evidence for Tyr B16 oxidation through ABS derivatization is given below.

##### F*_B1_VNQHLCGSHLVE_B13_

The N-terminus of chain B, Phe B1, displays a mass increase consistent with the oxidation of Phe B1 to cyclohexadienone. The MS/MS analysis (Figure S3) of the sequence F*VNQHLCGSHLVE indicates the formal addition of one oxygen atom and loss of 2 hydrogens from Phe B1, i.e. a mass increase of 14 Da through the appearance of the following ions: y9^+^, which suggests that the sequence HLCGSHLVE is not oxidized, and b6^+^, indicating the oxidation of the sequence FVNQHL. In this sequence, HL can be excluded as an oxidation target because of the nature of y9^+^. Since in the sequence FVNQ, F is most sensitive to oxidation we conclude that oxidation targets Phe B1.

##### F**_B1_VNQHLCGSHLVEALYLVCGE_B21_

Phe B1 can be further oxidized to DOCH (Figure S4). The observed masses of the ions y17^++^-H_2_O and y17^+^ exclude any oxidation of other amino acids sensitive to oxidation in F**_B1_VNQHLCGSHLVEALYLVCGE_B21_. The ion y17^+^ with m/z 1899.82 shows an intensity which is about 80 % of that of b16^+^ with m/z 1898.75; thus, it should not be considered the first isotope peak of b16^+^, which would be expected at m/z 1899.75.

#### ABS-Derivatized Glu-C Fragments

##### R_B22_GFFY^#^TPKT_B30_ and R_B22_GFFY^##^TPKT_B30_

Supplementary Figure S5 shows that Tyr B26 is converted to DOPA and/or DOCH, indicated by derivatization with one molecule of ABS (indicated with the symbol ^#^). The ions with m/z 361.28 and m/z 508.26, corresponding to b3^+^ and b4^+^, respectively, suggest that both Phe B24 and Phe B25 are not oxidized in this sequence. If for instance, Phe B24 (in the sequence RGF_B24_FYTPKT) had been oxidized, the b3^+^ ion would have been expected with m/z 377.28 (i.e. 361.28 + 16 Da). Instead, the ions b5^+^ and b6^+^ provide evidence that Tyr B26 is the target of oxidation. For additional evidence, supplementary Figure S6 displays the MS/MS spectrum of RGFFY^##^TPKT, derivatized with two molecules of ABS, as shown in the displayed structure. Here, the ions b3^+^ and b4^+^ are identical to the ones reported in Figure S5, although more intense. The ion with m/z 1037.29 corresponding to the ions b5^+^ and y5^+^ reported in the inset of Figure S6, confirm that the original Tyr contains two ABS molecules. We had realized in the past (19,20), that derivatization with ABS can lead to the incorporation of one or two molecules of ABS into the final benzoxazole product (see scheme 1), depending on the availability of ammonia, resulting in competition of ammonia and ABS for the 6-position in benzoxazole. In addition to that, it seems that the competition between ammonia and ABS depends on the steric hindrance of the peptide which is derivatized: i.e., derivatization with two ABS molecules of sterically less accessible DOPA and DOCH can be kinetically less favorable than that of more exposed residues. It must be noticed that the sequence R_B22_GFFYTPKT_B30_, depicted in Figures S5 and S6, does not necessarily belong to the same B chain: in other words, the sequence derivatized with one ABS molecule could be involved in the formation of HMWO, while the doubly ABS-derivatized sequence can actually be present in the monomer as well, although oxidized, and subsequently be derivatized with two ABS molecules.

#### Oxidized Fragments

##### F*_B1_VNQHLCG_B8_

Phe B1 is oxidized to hydroxylated Phe, indicated through the MS/MS data displayed in Figure S7: the ion y7^+^ shows that none of the amino acids in the sequence C-terminal to Phe B1, VNQHLCG, is oxidized. Therefore, the ion b5^+^ confirms oxidation of Phe B1. In fact, Phe hydroxylation can occur in positions ortho, meta and para; however, only the latter would lead to Tyr.

##### E_B21_RGF**FYTPKT_B30_

The MS/MS data displayed in Figure S8 are consistent with the oxidation of Phe B24 to DOCH. The ions b4^+^ and y6^+^ are fundamental to confirm the oxidation of Phe B24 since the first indicates the presence of the sequence ERGF**, and the second, suggests that the sequence FYTPKT is not oxidized. Further confirmation of the chemical structure in which Phe B24 is doubly oxidized arises from the detection of the internal fragment GF**FY with m/z 545.39, which can be produced during the analysis via specific pathways consistent with the mobile proton model (35).

##### F*_B1_VNQHLCGSHLVEAL_B15_

Phe B1 oxidation is also evident in the sequence F*VNQHLCGSHLVEAL, indicated by the MS/MS data displayed in Figure S9. Here it is sufficient to consider the ions y13^+^ and b14^++^. The ion y13^+^ indicates that the sequence NQHLCGS is not modified. On the other hand b14^++^ indicates oxidation in the sequence FVNQHLCGSHLVEA. By exclusion, this limits oxidation to the N-terminal subsequence FV, where F represents the most oxidation-sensitive amino acid.

#### ABS-Derivatized Fragments

##### A_B14_LY^#^LVC _B19_

We further corroborated the formation of DOPA through derivatization of the oxidized peptide ALY*LVC with ABS (Figure S10). The only possible site for derivatization in this peptide is an oxidized Tyr residue, since ABS derivatization requires the presence of DOPA (which during the derivatization is oxidized with K_3_Fe(CN)_6_ to DOCH).

##### S_A12_LY^#^QLE_A17_ and S_A12_LY^##^QLE _A17_

The MS spectra presented in Figures S11 and S12 provide evidence for the ABS derivatization of Tyr A14 within the sequence SLYQLE. Successful derivatization with one and two molecules of ABS indicates oxidation of Tyr A14 to either DOPA or DOCH. In both figures the ions b3^+^ with m/z 560.11 and 730.07, respectively and, the ion y4^+^ with m/z 748.39 and 918.15, respectively, indicate derivatization of the original Tyr residue with one and two molecules of ABS.

#### Non-oxidized Fragments

Two types of non-oxidized fragments were detected in the control (Table II). They are likely generated during production or storage as a consequence of low traces of transition metals. Instead, 14 non-oxidized fragments were detected as a result of MCO, summarized in the last column of Table II. The MS/MS data of all these fragments are reported in the Supplementary Material (Figures S13-S27).

#### Covalent Cross-Links

**Fig. 6:**
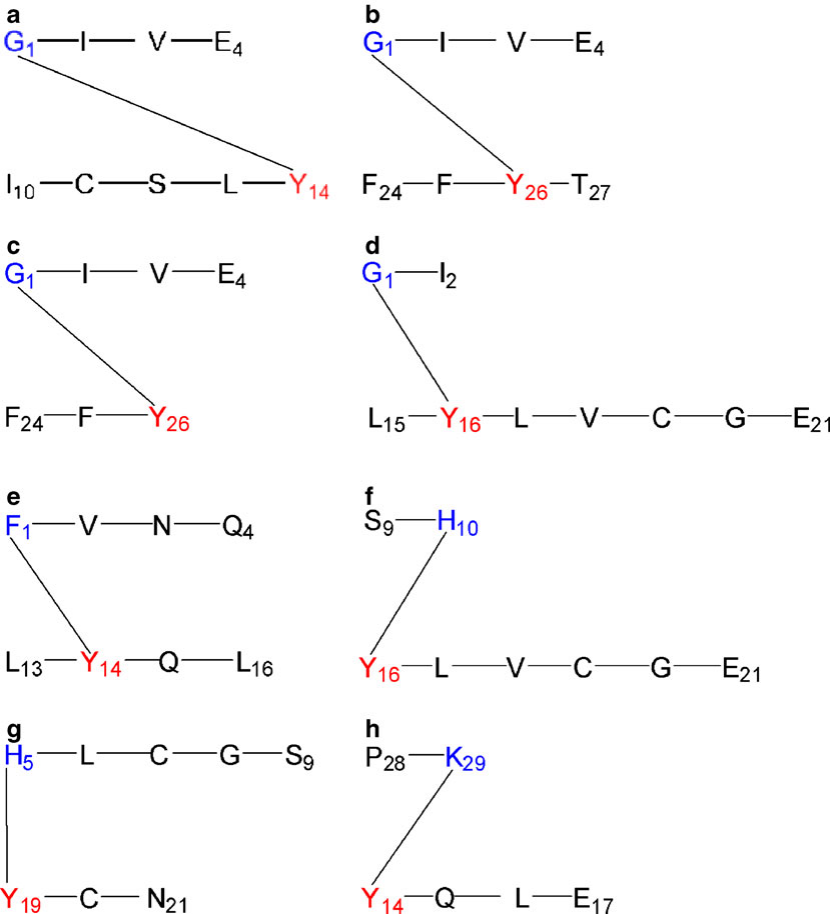
Identified inter-/intra-molecular cross-links measured after MCO of insulin, between (**a**) Gly A1-Tyr A14, (**b**, **c**) Gly A1-Tyr B26, (**d**) Gly A1-Tyr B16, (**e**) Phe B1-Tyr A14, (**f**) His B10-Tyr B16, (**g**) His B5-Tyr A19, (**h**) Lys B29-Tyr A14. In blue are shown the amino acids with nucleophilic properties and in red the amino acids oxidized to DOCH, which are Michael acceptor for nucleophilic addition. Further details are given in Fig. 7 and in the main text in the section “*Covalent Cross-Links”.*

This section focuses on the identification of covalent cross-links by MS/MS analysis. Figure 6 summarizes all the covalent cross-links detected as a result of the MCO of insulin.

##### Gly A1 –Tyr A14 Cross-Link

**Fig. 7:**
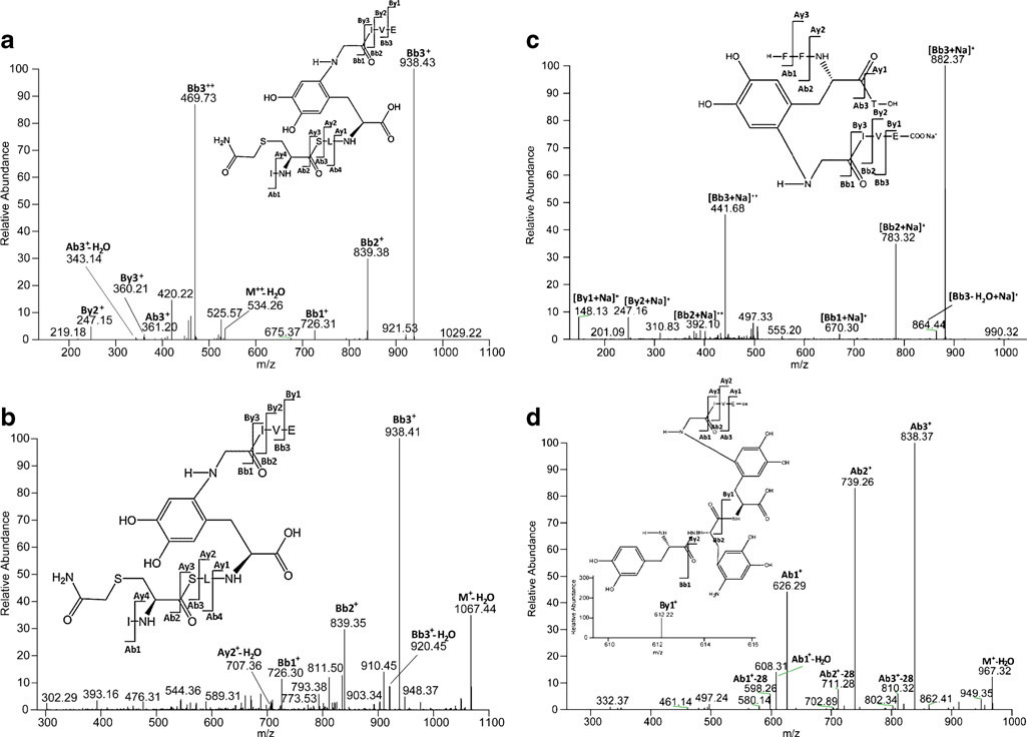

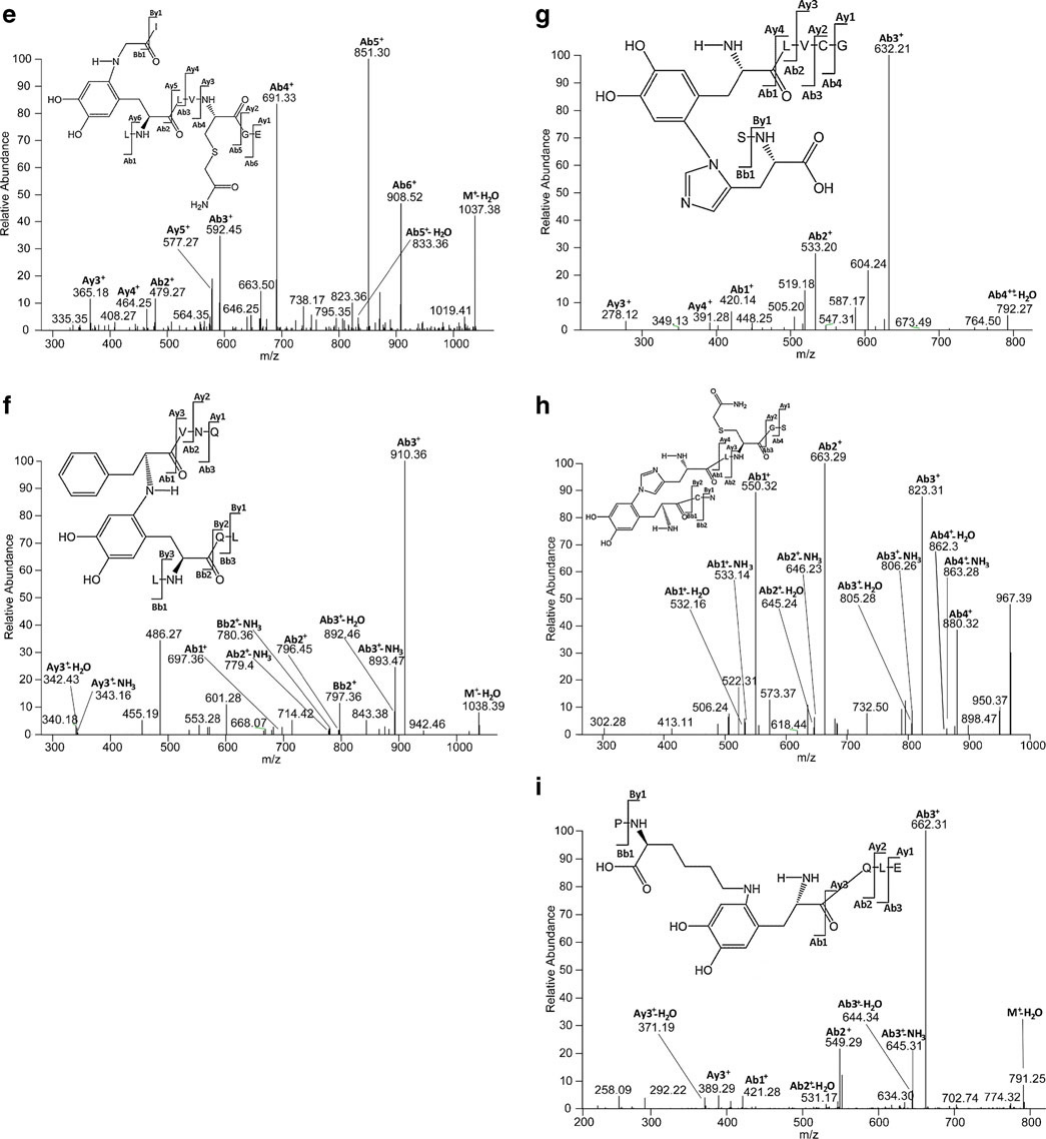
MS/MS of (**a**, **b**) sequence GIVE cross-linked through the glycine A1 to the sequence ICSLY, (**c**) sequence GIVE cross-linked through the glycine A1 to the sequence FFYT, (**d**) sequence GIVE cross-linked through the glycine A1 to the sequence FFY, (**e**) sequence GI cross-linked through the glycine A1 to the sequence LYLVCGE, (**f**) sequence FVNQ cross-linked through the phenylalanine B1 to the sequence LYQL, (**g**) sequence SH cross-linked through the histidine B10 to the sequence YLVCG, (**h**) sequence HLCGS cross-linked through the histidine B5 to the sequence YCN, (**i**) sequence PK cross-linked through the lysine B29 to the sequence YQLE. The fragments have been obtained after reduction (DTT), alkylation (IAM), ABS-derivatization and digestion (Glu-C) of oxidized insulin. Further explanation can be found in the text.

Figure 7a displays the MS/MS data consistent with a cross-link between Gly A1 and Tyr A14. The ions Bb1^+^, By3^+^ and Ab3^+^ are the most relevant ones to confirm the cross-link between Gly A1 and Tyr A14. In particular Bb1^+^ shows that the sequence ICSLY is covalently attached to Gly. The ion By3^+^ indicates that the sequence IVE is not involved in the cross-link and, therefore, can be dissociated during the MS/MS analysis since it is connected to Gly A1 only through a peptide bond (36). Finally, the ion Ab3^+^ provides evidence that ICS is not involved in the cross-link. In the structure provided in Fig. 7a, the covalent attachment of Gly A1 to Tyr A14 is representatively shown for the 6 position of the original DOPA (or DOCH) product. While, in principle, Michael addition is possible also at positions 1 and 2, position 6 is sterically less hindered, and that is why we selected to represent the adduct formation at position 6. However, we have currently no evidence for this regioselectivity. Further confirmation of the structure reported in Fig. 7a was obtained through the spectrum displayed in Fig. 7b, where the ion M^+^-H_2_O was detected.

##### Gly A1—Tyr B 26 Cross-Link

Figure 7c represents the cross-link of Gly A1 of the sequence GIVE to Tyr B26. The mass spectra displayed in Fig. 7c represents sodium adducts of the respective peptide fragment(s), likely due to incomplete removal of buffer during dialysis. Therefore, all the m/z of the ions presented in Fig. 7c have an increased mass of 23 Da. The ion [By2 + Na]^+^, representing the singly charged sequence VE, suggests that the amino acids Val and Glu are not involved in the new covalent bond. If this sequence had been involved in the new covalent bond with Tyr, it would not have been possible to detect the ion [By2 + Na]^+^, since only dissociation of peptide bonds occurs during the low energy collision with the inert gas. [Bb2 + Na]^+^ indicates that the sequence GI is covalently bound to the sequence FFY. We excluded the possibility of DOPA as the product of one of the Phe residues, since in such case the ion [Bb2 + Na]^+^ would have a mass increase (relative to the expected mass of the fragment of unmodified insulin) larger than 16 Da (the oxidation of Phe to DOPA and DOCH requires two oxygen atoms); if one of the Phe residues were exclusively oxidized to Tyr, the ion [Bb2 + Na]^+^ would have the same molecular weight as the one detected in this MS/MS spectrum. In such case, however, it would not be possible to observe Michael addition, since the new Tyr residue (generated from the mono-oxidation of Phe) is not a Michael acceptor unless it is further oxidized to quinone.

In Fig. 7d the original sequence FFY shows additional oxidation of both Phe residues (in contrast to Fig. 7c, which shows the native Phe residues in FFY): one of them to DOPA and the second one to a 6-amino substituted DOPA, likely originating from Michael addition of ammonia to a DOPA oxidation product. The ions By1^+^, with m/z 612.22, displayed in the inset, and Ab1^+^, with m/z 626.29, unequivocally indicate that the covalent bond is between Gly and Tyr: By1^+^ represents the singly charged sequence Y-GIVE and Ab1^+^ corresponds to the singly charged sequence FFY-G. The combination of both demonstrates that the new covalent bond, not dissociable during MS/MS analysis, is located between Tyr and Gly. Importantly, our results show that predominantly Tyr oxidation products serve as Michael acceptors for cross-link formation during the MCO of insulin (see also below). There are several possible rationales for this behavior, including protein conformation and the respective yields of Tyr and Phe oxidation products: the oxidation of Phe to DOCH requires one additional oxidation step compared with that of Tyr to DOCH, suggesting that DOCH formation from Tyr is kinetically favorable.

##### Gly A1—Tyr B16 Cross-Link

Figure 7e displays MS/MS data consistent with a covalent cross-link between Gly A1 and Tyr B16. The most relevant ion in this figure is Ab2^+^ with m/z 479.27, which demonstrates that the sequence GI is covalently bound to LY. Since Ile and Leu do not contain functional groups amenable to Michael addition, we conclude that the covalent bond is formed between Gly and Tyr.

##### Phe B1—Tyr A14 Cross-Link

Phe B1 is not only a possible target for oxidation; the free N-terminal amino group can react via Michael addition with DOCH. The spectrum depicted in Fig. 7f shows the relevant ions consistent with a cross-link between Phe B1 and Tyr A14: Ab1^+^ with m/z 697.36 shows the existence of a peptide containing the following amino acids: Phe, Tyr, Gln and two Leu, plus the molecular weight of one oxygen atom. However considering only the ion Ab1^+^, it is not possible to localize the oxygen addition and to characterize the nature of the cross-link. The ion Bb2^+^, with m/z 797.36, indicates that the sequence FVNQ is connected to the sequence LY. The ion Ay3^+^ -NH_3_, with m/z 343.16, excludes any covalent cross-link of the sequence VNQ. In the sequence LY only Tyr oxidation can lead to a Michael acceptor for the amino terminal of Phe B1, which suggests a cross-link such as depicted in Fig. 7f.

##### His B10—Tyr B16 Cross-Link

Figure 7g displays a cross-link between His B10 of the sequence SH and Tyr B16 of the sequence YLVCG. The relevant ions in this spectrum are Ab1^+^ and Ay4^+^. Ion Ab1^+^, with m/z 420.14, suggests that the sequence SH is covalently bound to the original Tyr residue (after oxidation of Tyr to DOCH). The deprotonated imidazole nitrogen of His is an appropriate nucleophile for Michael addition, demonstrated for example for His addition to dehydroalanine (36).

##### His B5—Tyr A19 Cross-Link

Figure 7h displays a cross-link between His B5 of the sequence HLCGS and Tyr B16 of the sequence YCN and the MS/MS data supporting this assignment. We note that the ion Ab1^+^-NH_3_ shows an intensity which is about 80 % of that of Ab1-H_2_O. Thus, the ion with m/z 533.14 should not be considered the second isotope of Ab1^+^-H_2_O, suggesting that the N-terminal amino group of the His is free and not involved in the new covalent bond.

##### Lys B29—Tyr A14 Cross-Link

Figure 7i represents the cross-link between Lys B29 and Tyr A14. The relevant ion in the spectra is Ab1^+^, since it localizes the cross-link to Tyr and the amino acids Pro and Lys in the sequence PK. Because the MS/MS data in Fig. 7i shows no B ions, we cannot conclude whether the cross-link involves the side chain amino group of Lys or the amino group of Pro. The latter would require that non-oxidative fragmentation between Thr B27 and Pro B28 occurs prior to cross-linking; the side chain amino group of Lys B29 represents a good nucleophile and it can therefore be assumed that at least some of the cross-links are formed through the reaction of Tyr A14 with Lys B29.

## Discussion

We performed a detailed mass spectrometric analysis of insulin in order to elucidate the mechanisms and amino acid residues involved in MCO and MCO-mediated aggregation. The oxidation of Phe and Tyr leads to the formation of catechol structures that can be further oxidized to DOCH. These oxidation products serve as Michael acceptors for cross-links with several nucleophiles present in the insulin sequence. Formation of dityrosine, as a potential structure involved in the cross-linking of insulin, was not detected, neither with fluorescence measurement (see material and methods), nor with the help of MassMatrix software. This is not surprising since it has been reported that only the hydrogen peroxide/copper system is capable of inducing dityrosine formation (37). Schiff base formation could potentially be in competition with Michael addition, nonetheless such cross-links can be reversed relatively easily and based on our results does not appear to be the main mechanism of insulin aggregation. Changes in the primary structure, as reported in this paper, may alter the biological activity of insulin with severe consequences on the glycemic control. Furthermore, changes in the secondary, tertiary and quaternary structure as a consequence of oxidation, as reported by us before (13), may generate new repetitive epitopes or open the access to hidden epitopes that could contribute to the formation of immunogenic products.

In addition to the characterization and localization of oxidation products, we provided quantitative data on the amount of fragments and oxidized monomers. An important feature of insulin exposed to MCO is the appearance of fragments, which increases the number of polypeptide nucleophiles available for Michael addition. At this point, we do not know whether the observed insulin fragmentation precedes or follows cross-link formation. Furthermore, it is unknown whether Glu-C might have a different specificity towards oxidized protein. The actual mechanism of cleavage is currently unknown, but we can exclude the classical oxidative cleavage (38) since we did not detect the α,β-dicarbonyl products expected for such a mechanism. Instead, all non-specific cleavages appear to involve a hydrolytic cleavage of the respective peptide bonds. It may be possible that the addition of Cu^2+^ and L-ascorbate promote the formation of peptide-metal complexes with hydrolytic activity, such as described for the cleavage of amides through Cu^2+^ (39). Surprisingly, most of the non-specific peptide fragments we detected were already reported by other authors, e.g. after the oxidation of glycated insulin by Fenton chemistry (9,33); however, Guedes *et al.*(33) interpreted the cleavage mechanism as oxidative cleavage, which is *inconsistent* with the intact C- and N-termini of the detected peptide fragments.

Through MCO experiments at various pH, and in the presence of ammonia, we are able to confirm that Michael addition between insulin chains causes covalent aggregation. In the presence of ammonia, which competes for Michael addition to DOCH, aggregation was almost completely inhibited. Although such experimental conditions allow to prevent aggregation, we noted that the introduction of an amino group on oxidized Tyr or Phe might still have consequences for the immunogenicity of this small polypeptide hormone.

Several proteins have been shown to form aggregates after exposure to Cu^2+^/L-ascorbic acid, such as recombinant SHa(29–231) prion protein (40), superoxide dismutase (41), monoclonal IgG2 (42), interferon alpha 2a (5,6) and interferon beta 1a (7). The results presented in this paper may serve as a model to rationalize the chemical mechanisms of aggregation during MCO of proteins in general. Although insulin lacks Trp and Met, which are also prone to oxidation (43,44) and are present in most other proteins, DOPA and DOCH should not be formed from oxidation of Trp or Met, thus they cannot act as electron acceptor for Michael addition. Since MCO was performed at pH 7.4, where insulin is present mainly in the hexameric state (45), the cross-links we measured are likely to be *inter*molecular cross-links, i.e. formed between different insulin molecules. As an example we calculated the distance of Gly A1 and Tyr A14 and Gly A1 and Tyr A19 within the same A-chain (using Swiss pdb viewer (46,47) and T6 human insulin at 1.0 Å resolution, 1MSO pdb file). An average of 18.5 Å was measured for a theoretical covalent bond between the amino group of Gly A1 and the carbon in ortho of the aromatic ring of Tyr A14, and an average of 8.3 Å for the amino group of Gly A1 and the carbon in ortho of the aromatic ring of Tyr A19, which seems to be too far for an *intra*molecular reaction (e.g., the length of the peptidic bond between Gly A1 and Leu A2 is 1.3 Å). We also noticed that Phe residues, oxidized to DOCH, were not involved in cross-links, nor were they derivatized with ABS. In both cases this could be due to low accessibility of Phe residues which might belong to chains B involved in the formation of HMWO, in which only DOCH originated from Tyr is accessible for ABS derivatization. Moreover, in the case of cross-links it could be speculated that the oxidation of Tyr to DOCH (which requires the addition of only one oxygen atom), is kinetically favorable over the oxidation of Phe to DOCH (which requires two oxidation steps). This can potentially lead to a depletion of the nucleophiles available in the insulin molecule.

## Conclusions

Oxidation is one important degradation process that proteins can undergo. In this work we highlighted the potential consequences of DOPA and DOCH formation and also illustrated how the knowledge of oxidation mechanisms can be utilized to investigate the mechanism of covalent aggregation. To this end, recombinant human insulin was used as a model: oxidation of aromatic amino acids residues, besides others, leads to α,β unsaturated carbonyl compounds, which are electron acceptors for Michael addition. These reactive groups, resulting from oxidation, not only can lead to covalent protein aggregation, as shown for insulin in this paper, but also may lead to cross-links between protein molecules and amino acids, which are typically present during cell culture and are often used as formulation excipients. This must be taken into consideration during production and formulation development of therapeutic proteins.

## Electronic supplementary material

Below is the link to the electronic supplementary material.

Esm 1 (DOC 2.78 mb)

## Abbreviations

ABI: ammonium bicarbonate
ABS: 4-(aminomethyl) benzenesulfonic acid
CB: sodium citrate buffer
DOCH: 2-amino-3-(3,4-dioxocyclohexa-1,5-dien-1-yl) propanoic acid
DOPA: 3,4-dihydroxyphenylalanine
DTT: dithiothreitol
EDTA: ethylenediaminetetraacetic acid
ESI-MS: electrospray ionization-mass spectrometry
FT-ICR MS: Fourier transform ion cyclotron resonance mass spectrometry
HMWO: high-molecular-weight oligomers
IAM: iodoacetamide
MCO: metal catalyzed oxidation
PB: sodium phosphate buffer
SEC: size exclusion chromatography
