# Supplementary material for: Chemical Modifications in Aggregates of Recombinant Human Insulin Induced by Metal-Catalyzed Oxidation: Covalent Cross-Linking via Michael Addition to Tyrosine Oxidation Products
Source: Pharm Res. 2012 May 10;29(8):2276–93. doi: 10.1007/s11095-012-0755-z (PMC3399080; doi:10.1007/s11095-012-0755-z)
Supplement: Supplementary file 1 — (DOC 2.78 mb) [file 11095_2012_755_MOESM1_ESM.doc]

**Supplementary Material**

In the supplementary material are reported all the MS/MS spectra of Oxidized Glu-C Fragments, ABS-Derivatized Glu-C Fragments, Oxidized Fragments, ABS-Derivatized Fragments and Non-oxidized Fragments. A complete list can be seen in table II in the main text. Description of Glu-C Fragments and Fragments, both oxidized and ABS-derivatized, is given in the main text in the section “Identification of Chemical Modifications by MS/MS Analysis of Reduced, Alkylated, ABS-Derivatized and Digested samples”. Here we define “fragments” as peptides resulting from at least one cleavage site that differs from the one expected from proteolytic digestion by Glu-C (i.e. after glutamic acid) and we define “Glu-C fragments” as peptides originating from expected proteolytic digestion by Glu-C.

**Oxidized Glu-C Fragments**

**A**

**B**

**C**

Figure S1


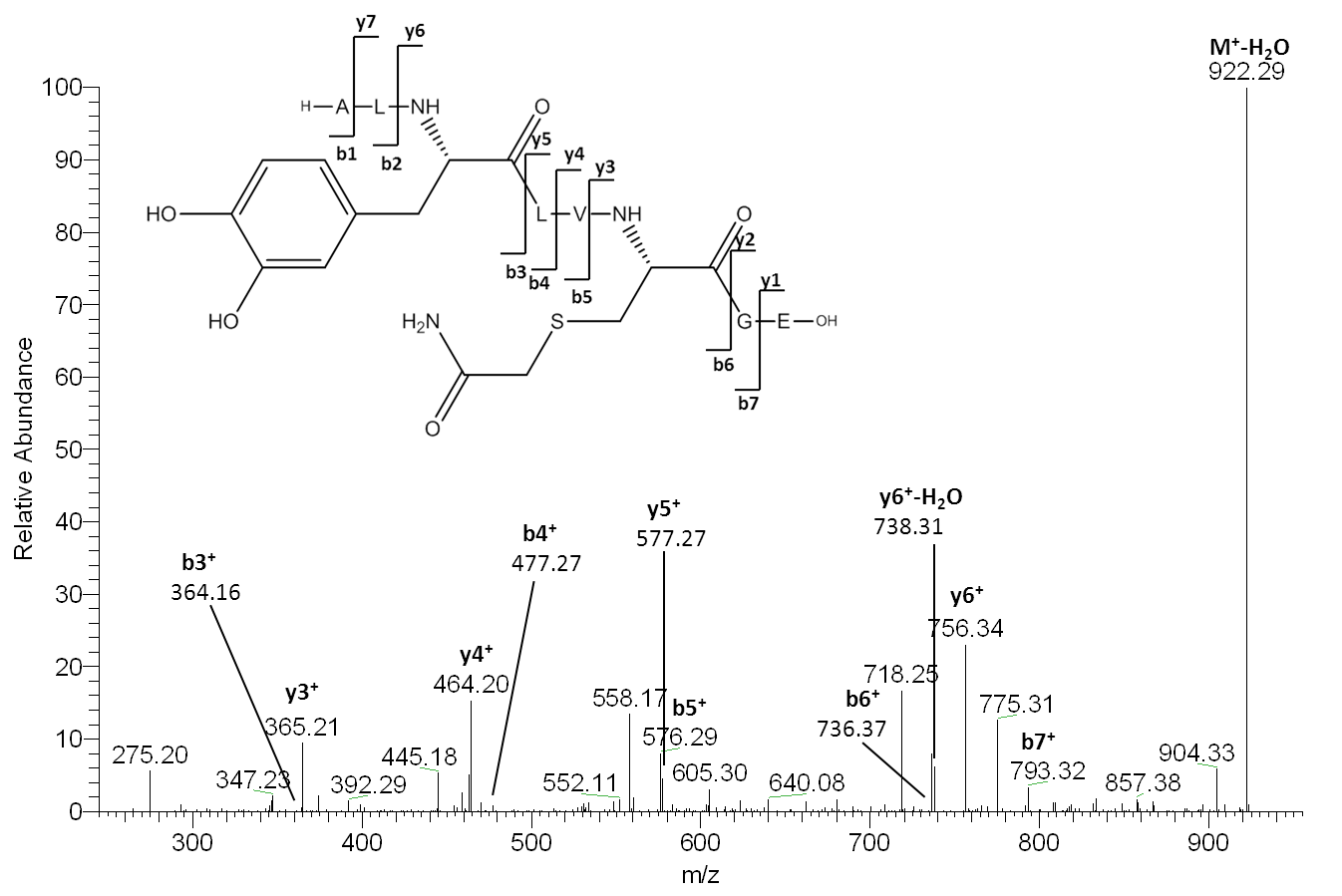


Figure S2


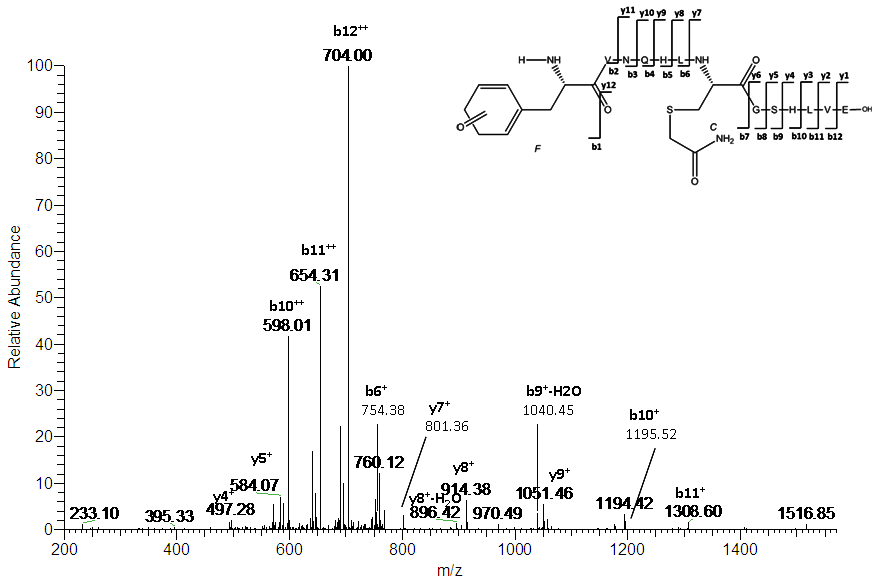


Figure S3


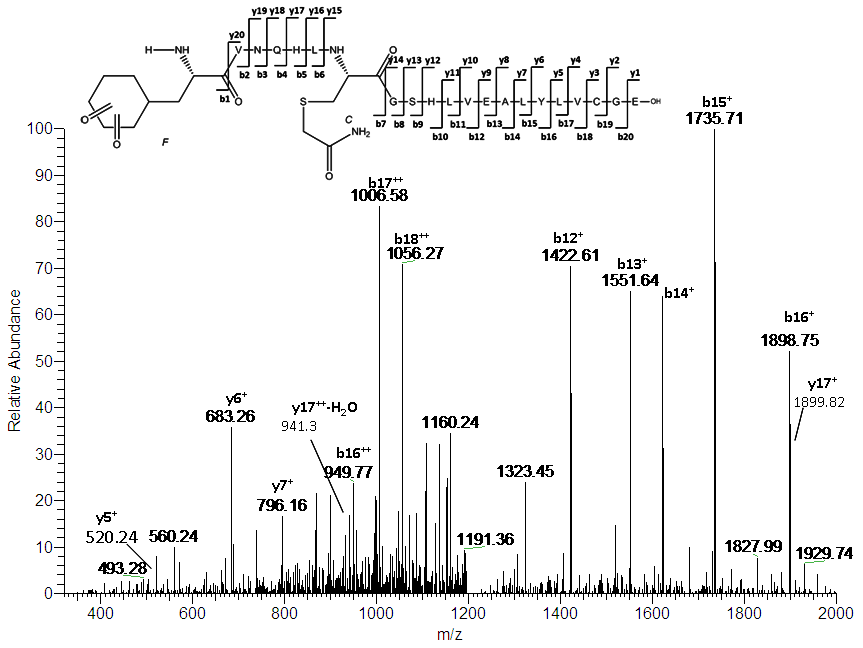


Figure S4

**ABS-Derivatized Glu-C Fragments**


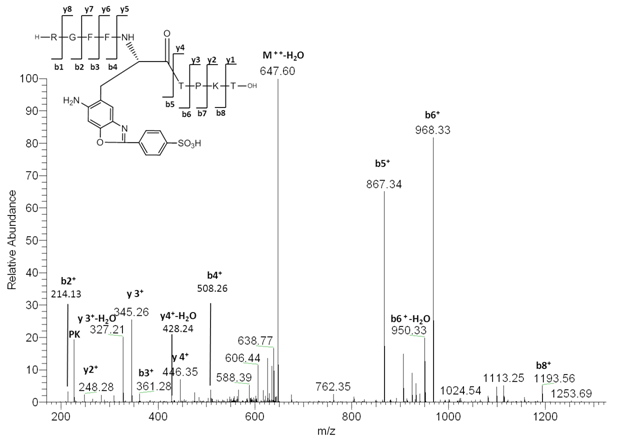


Figure S5


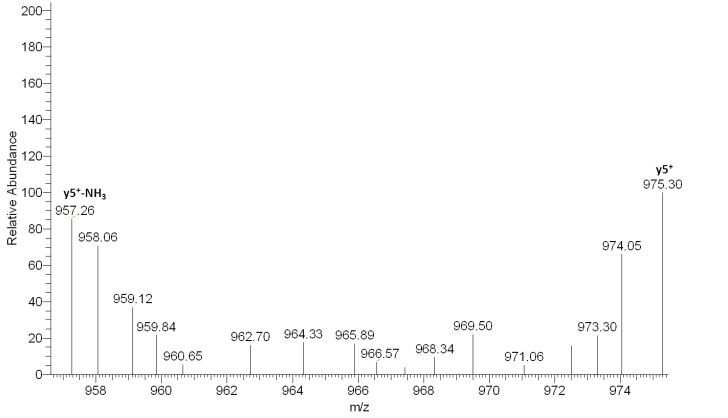

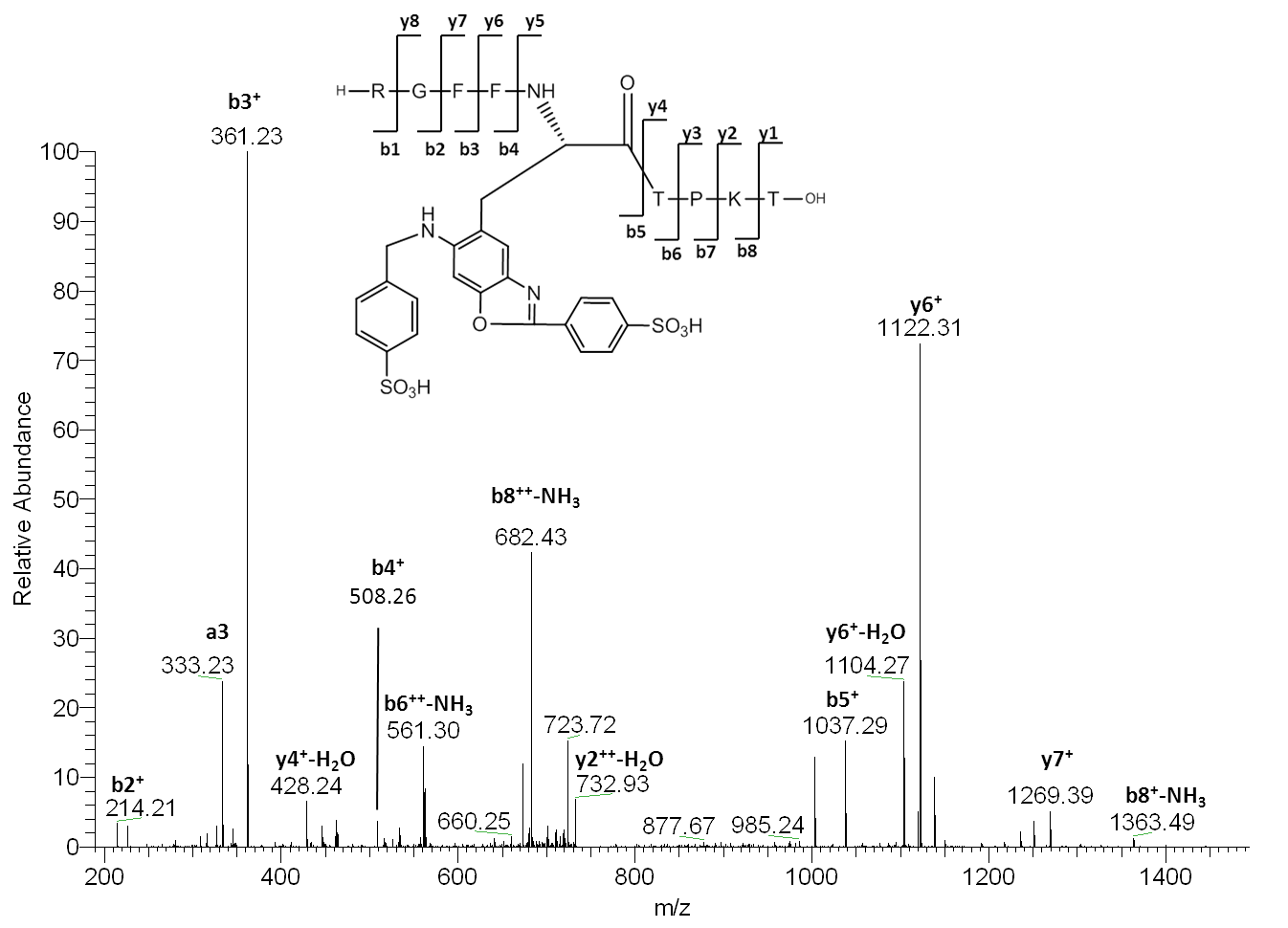


Figure S6

Oxidized Fragments


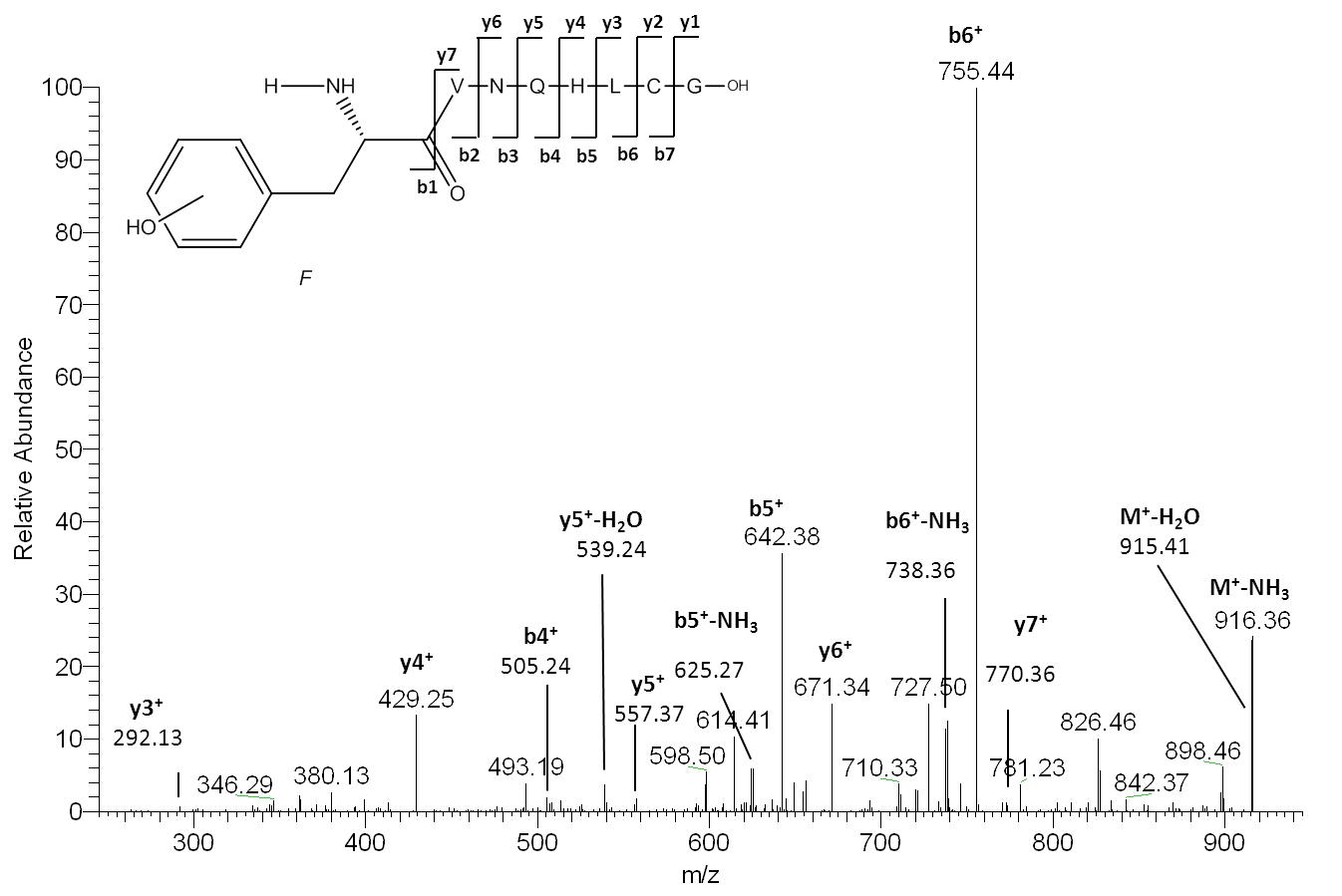


Figure S7


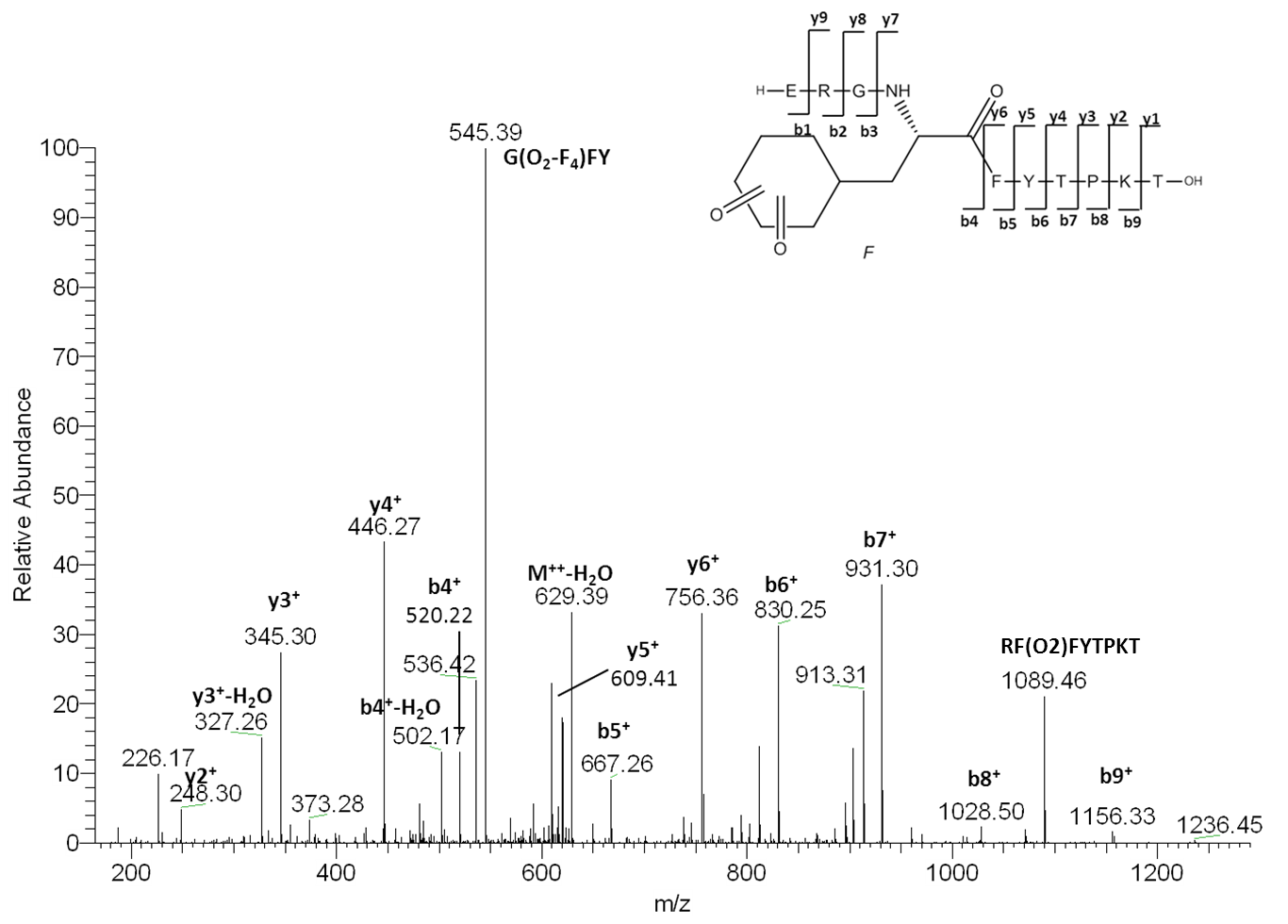


Figure S8


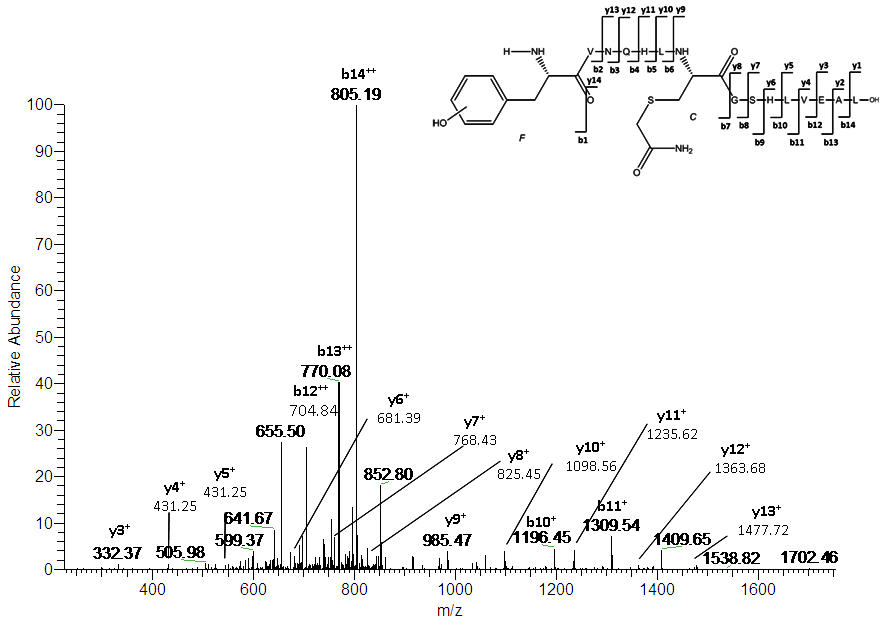


Figure S9

**ABS-Derivatized Fragments**


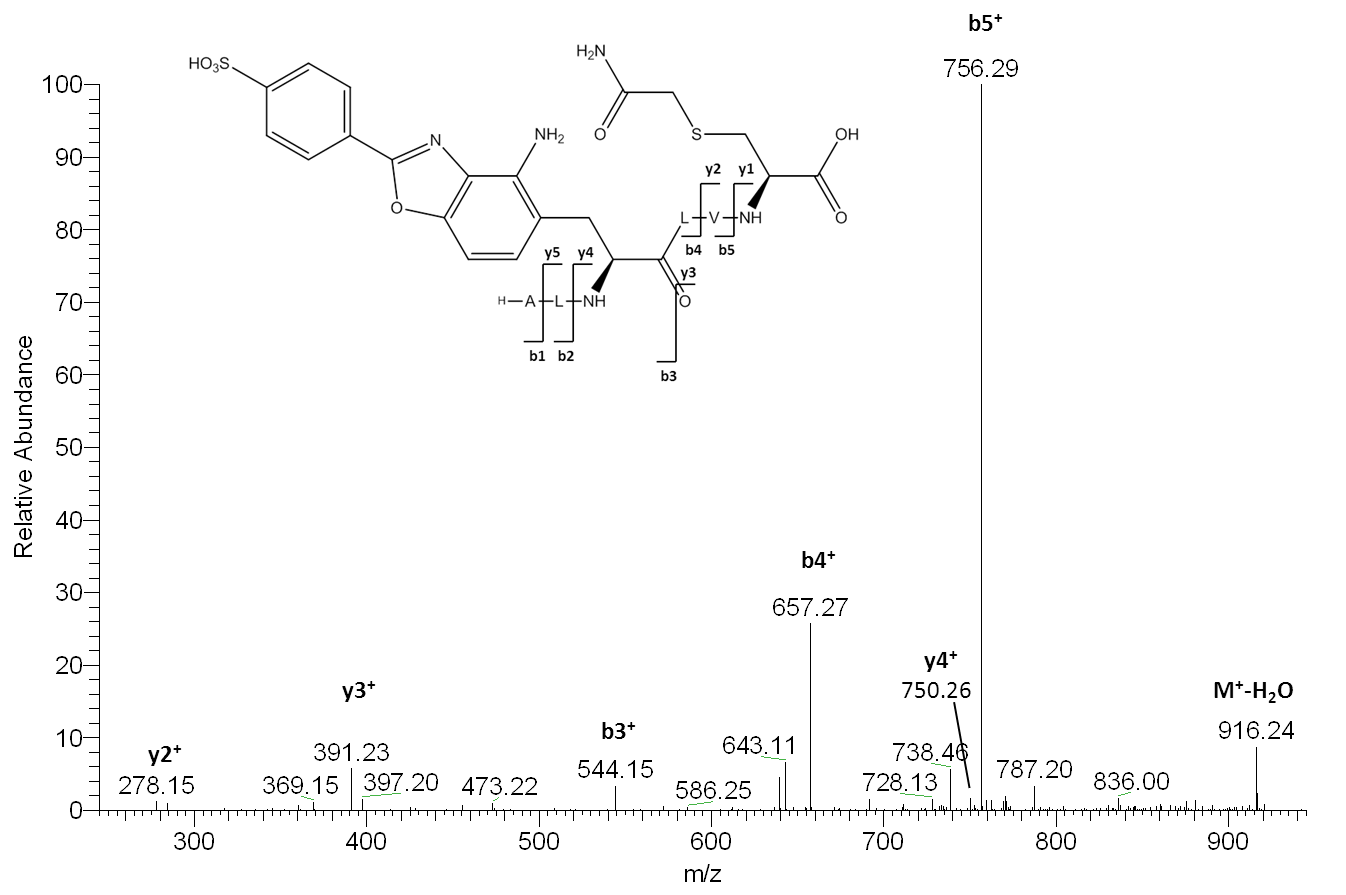


Figure S10

Figure S11


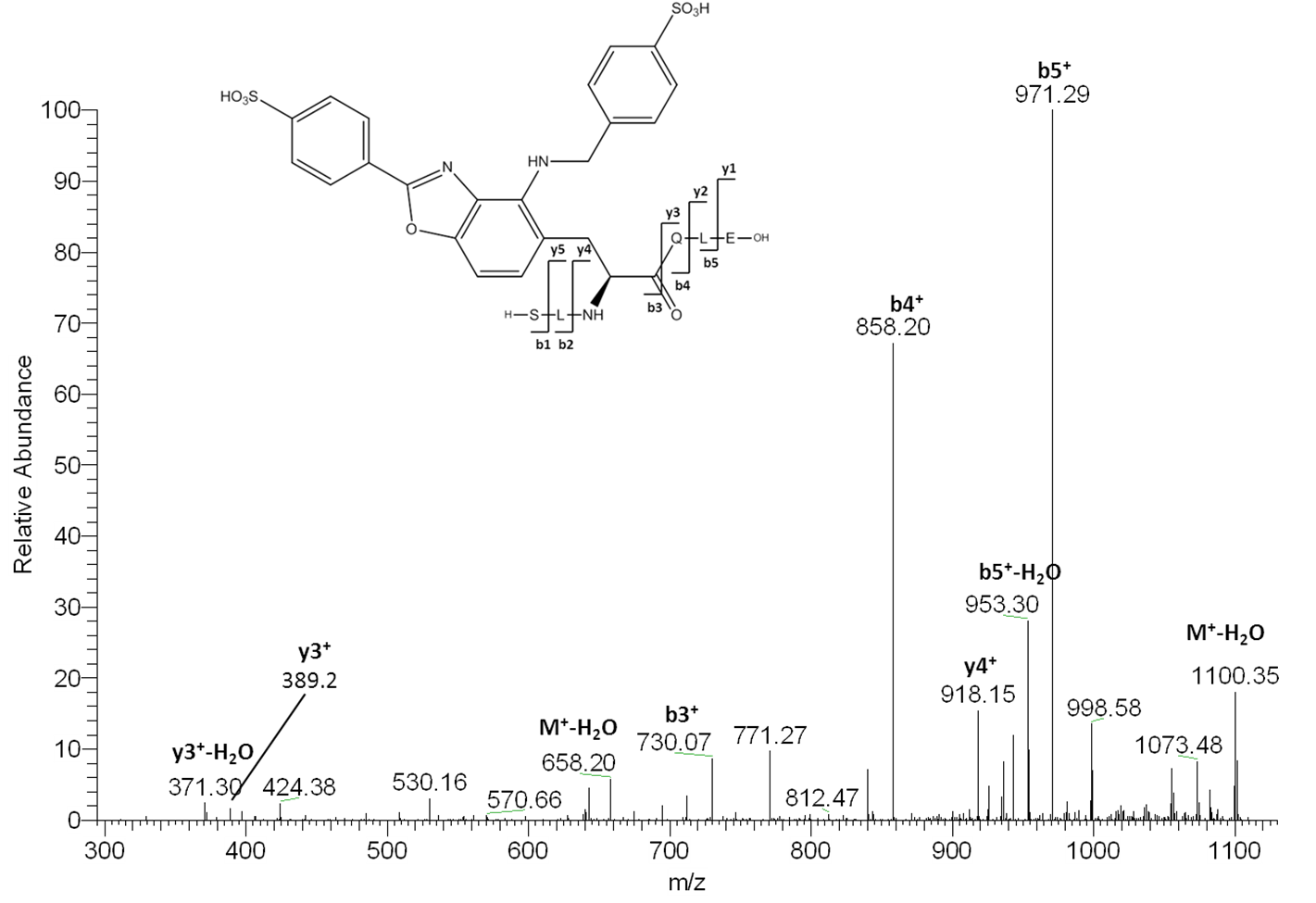


Figure S12

**Non-oxidized** **Fragments**


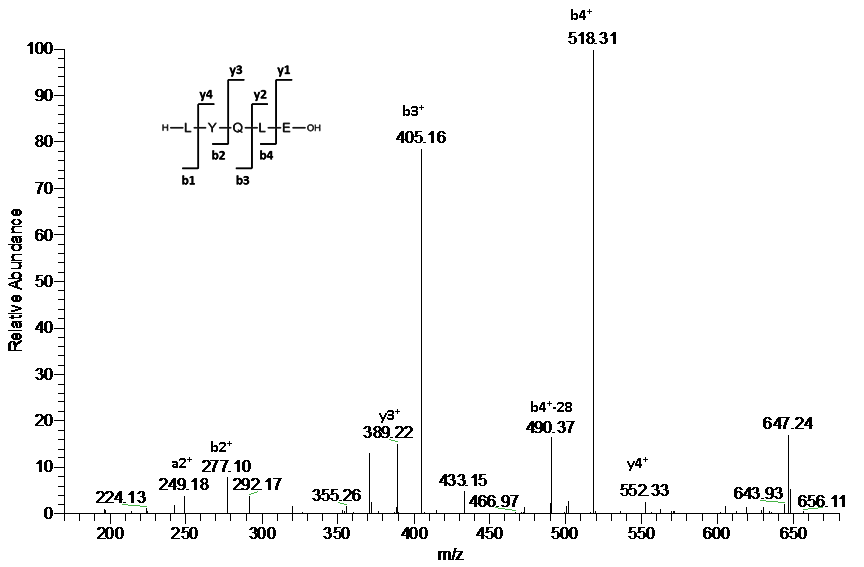


Figure S13


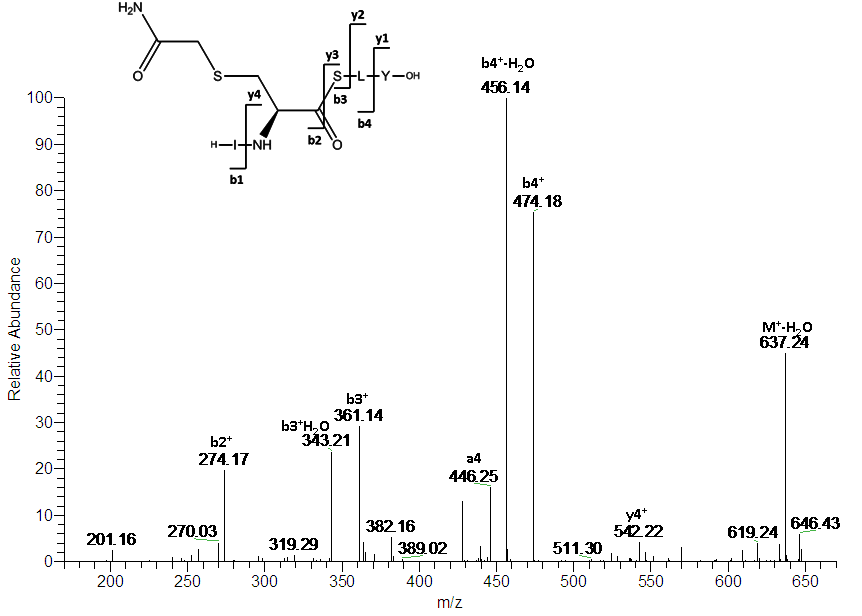


Figure S14


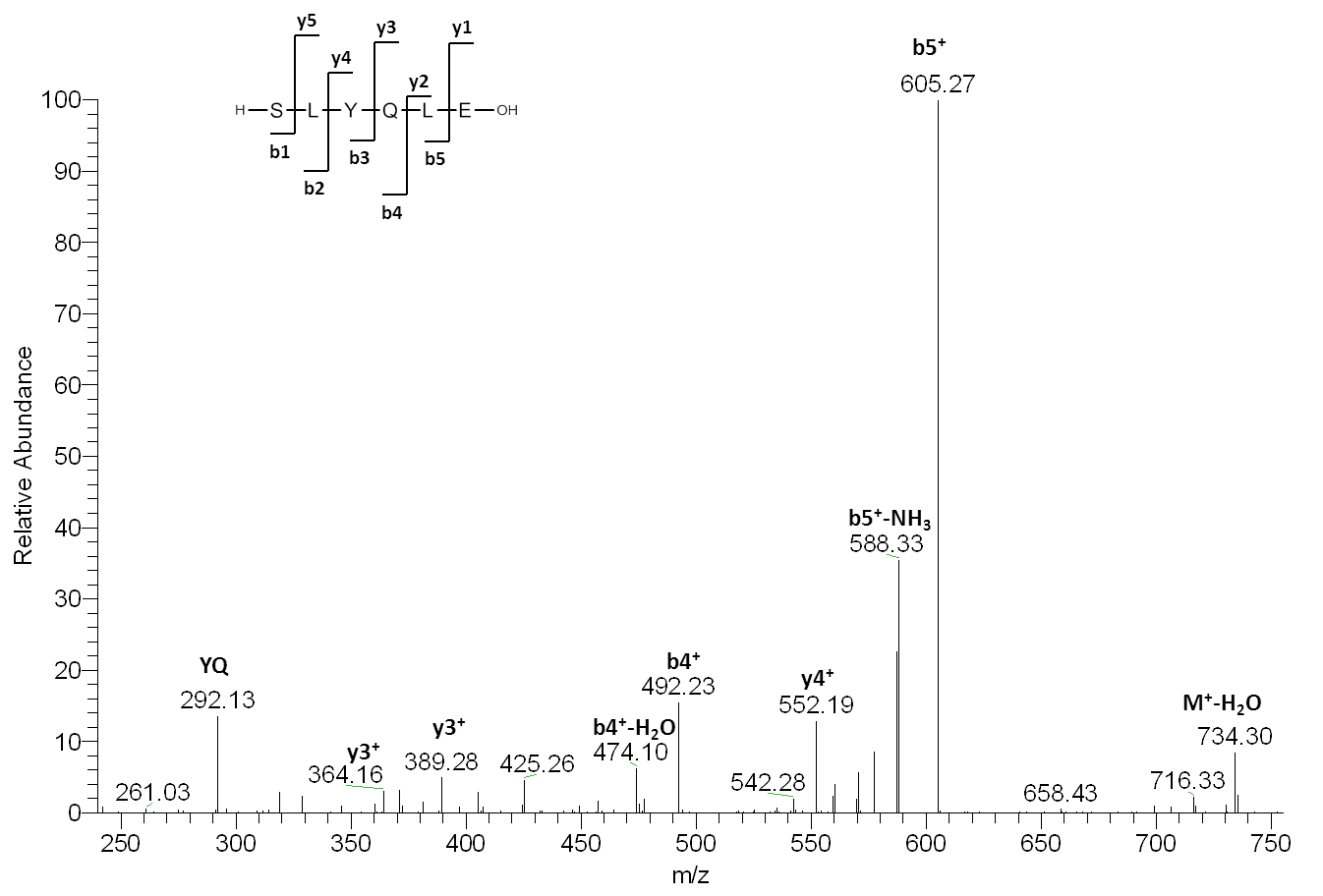


Figure S15


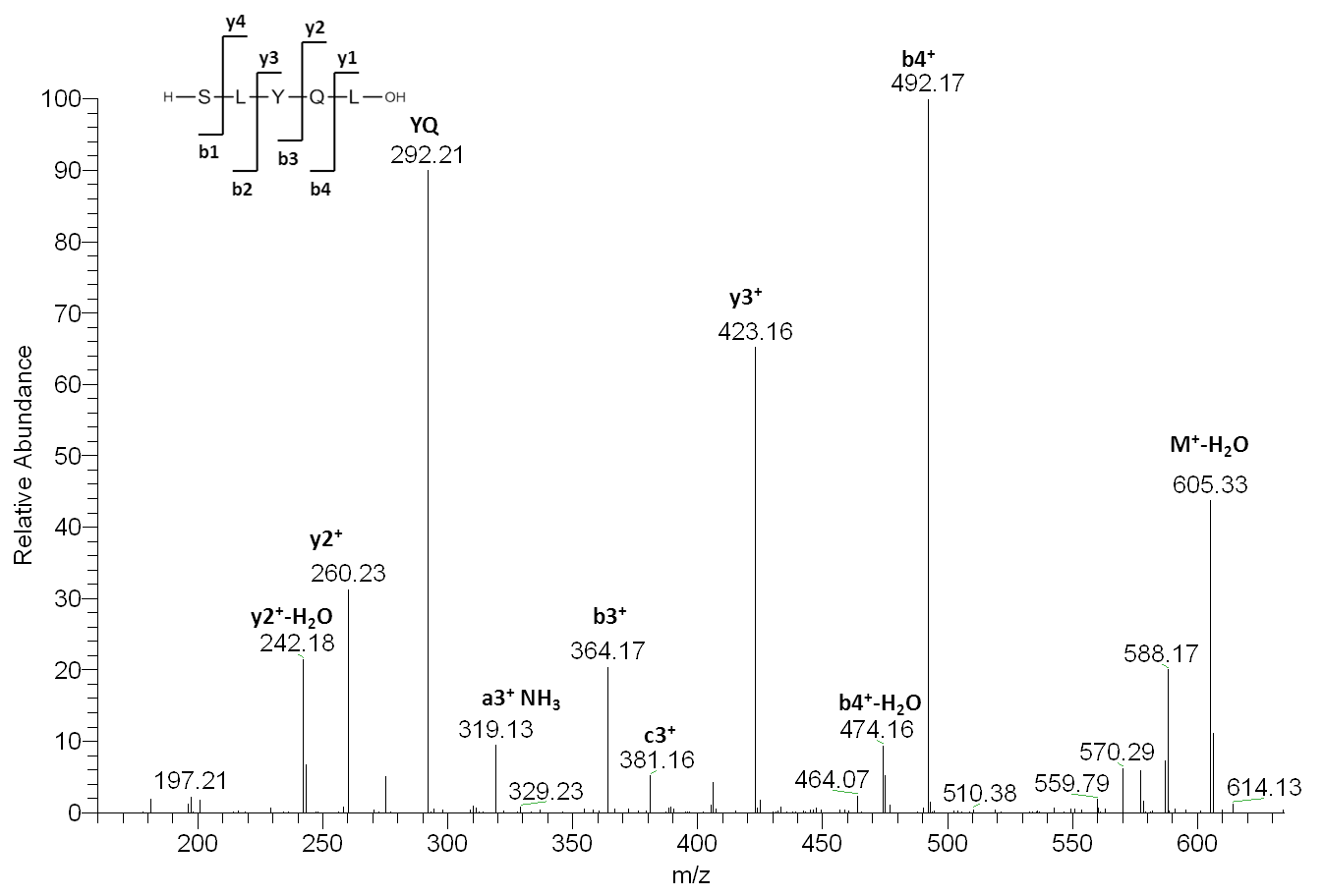


Figure S16


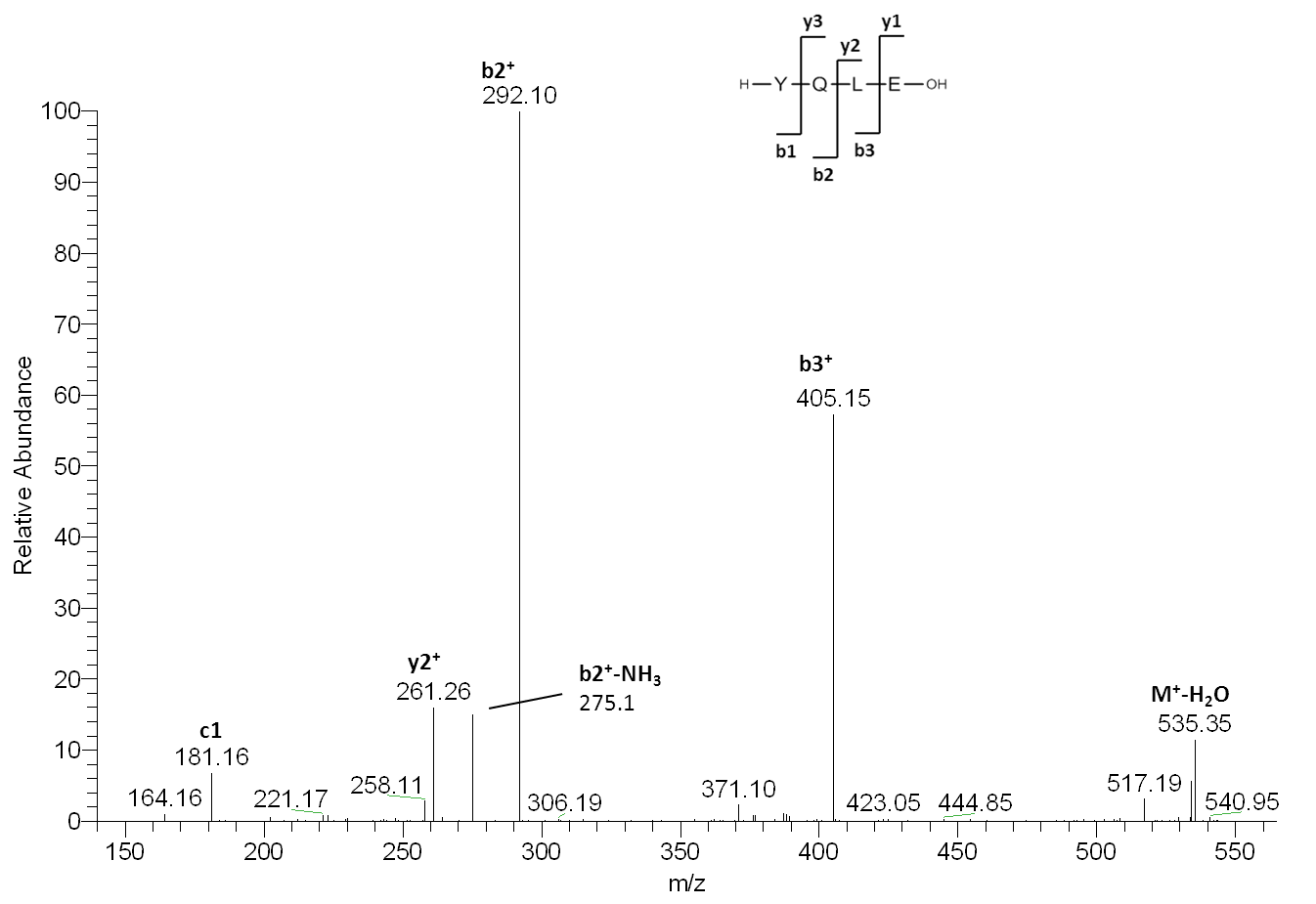


Figure S17


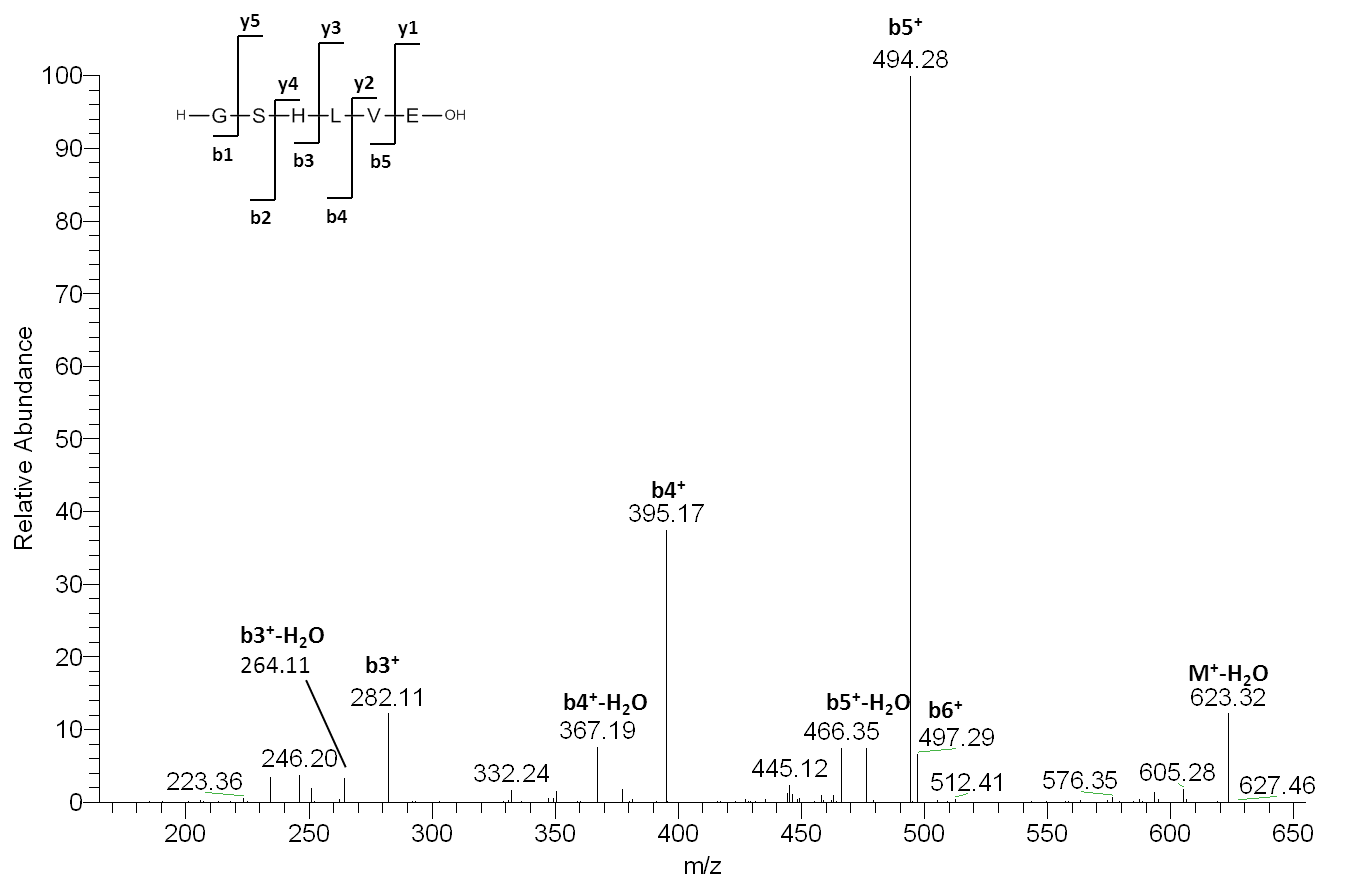


Figure S18


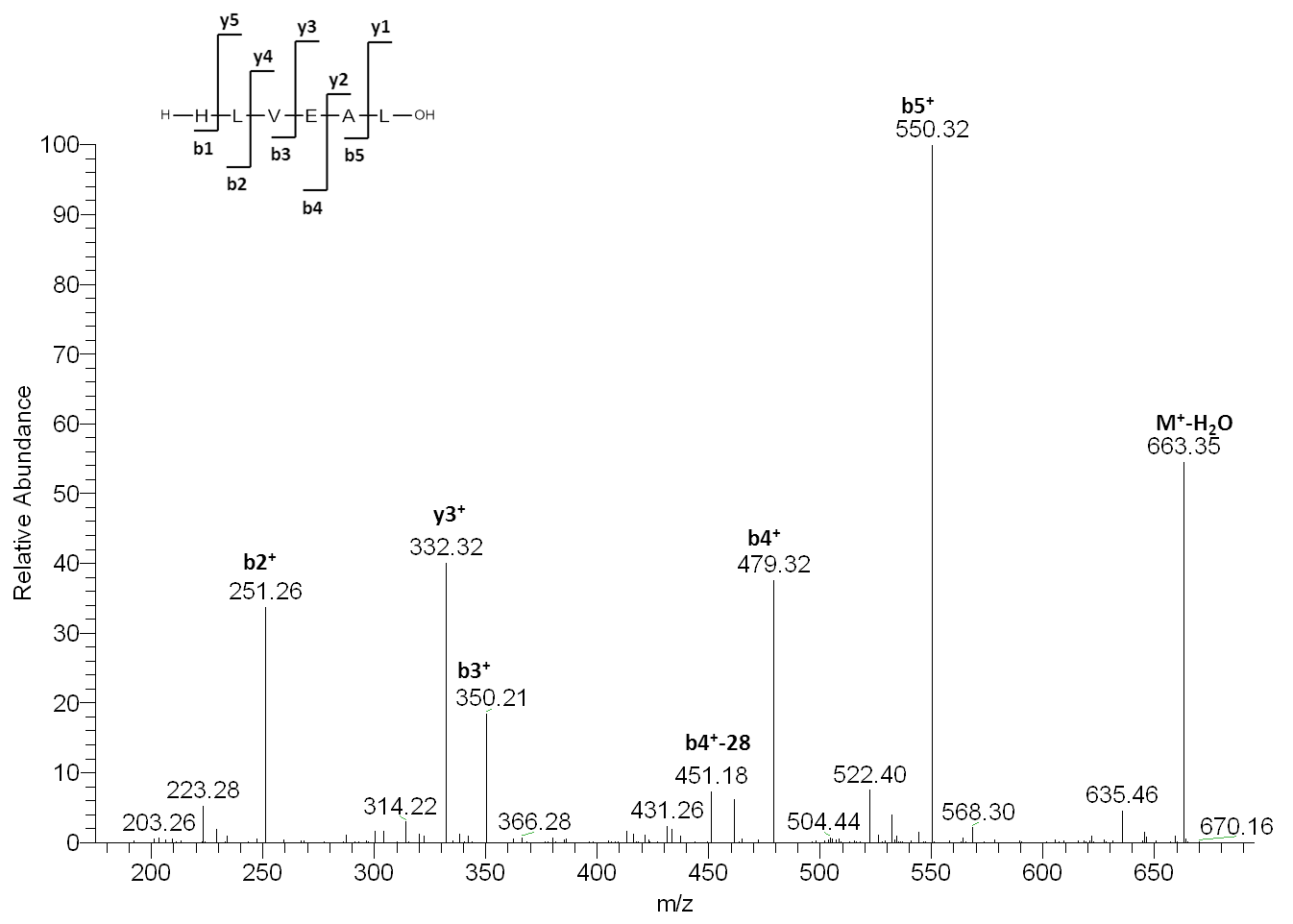


Figure S19


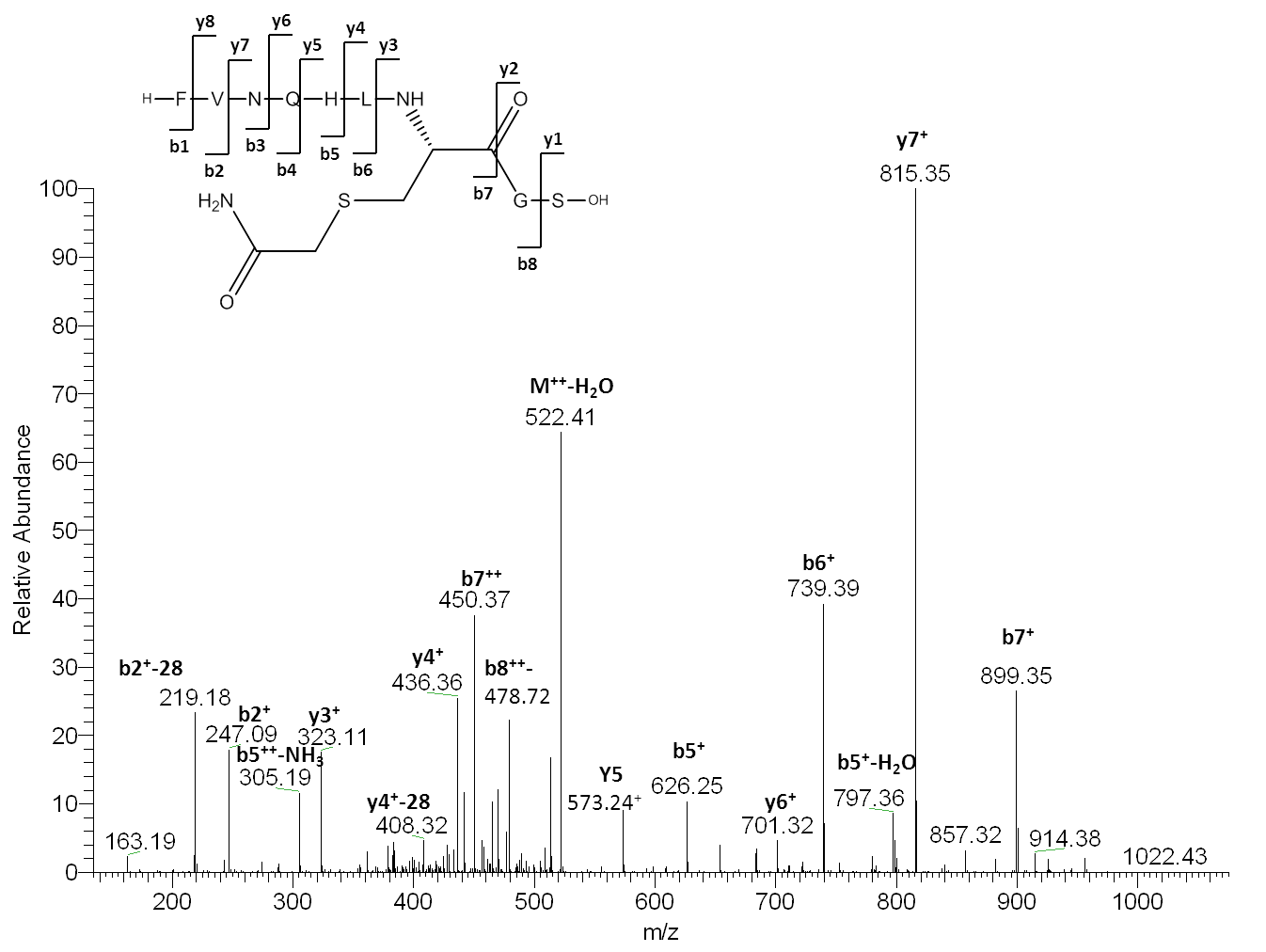


Figure S20


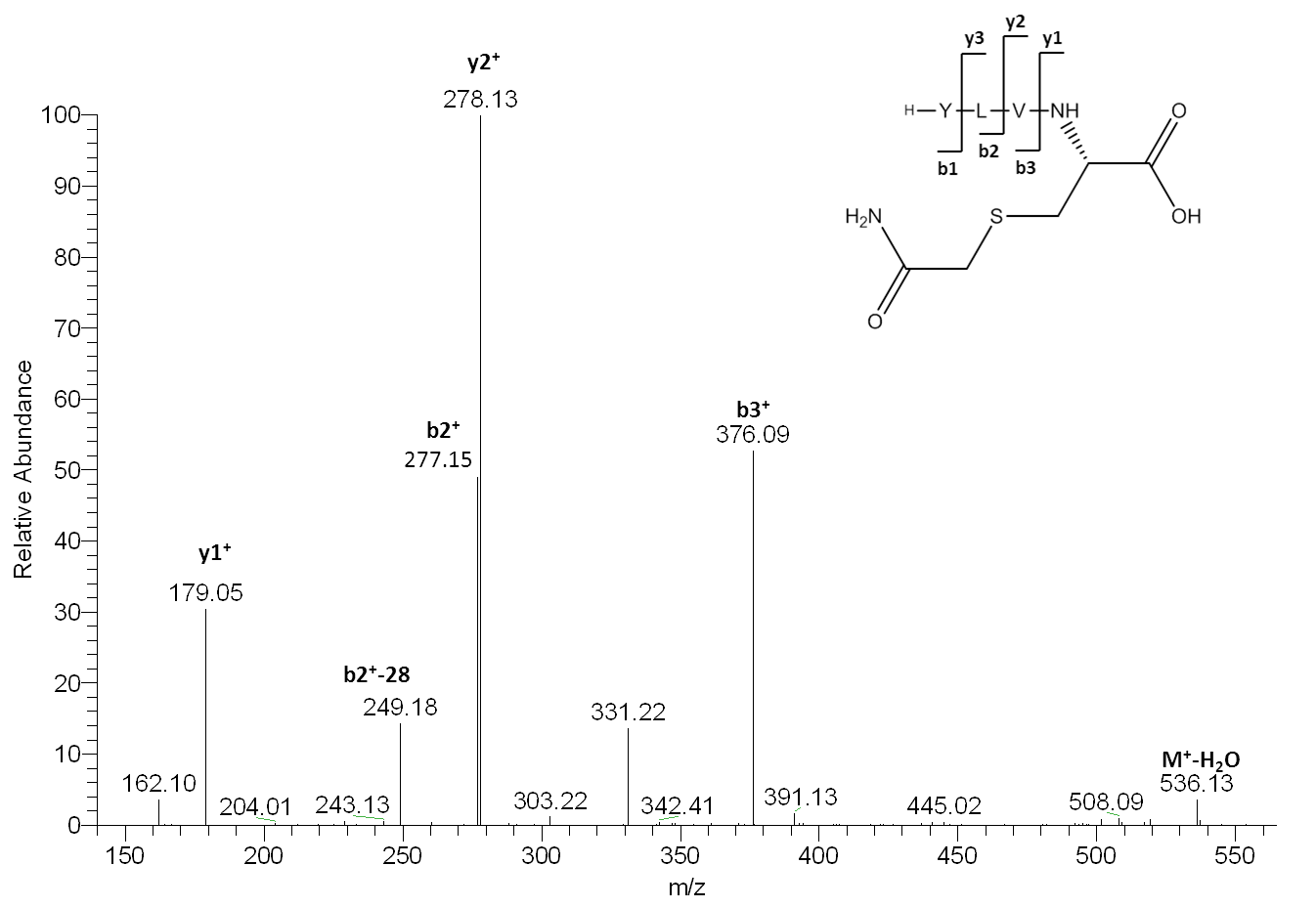


Figure S21

Figure S22


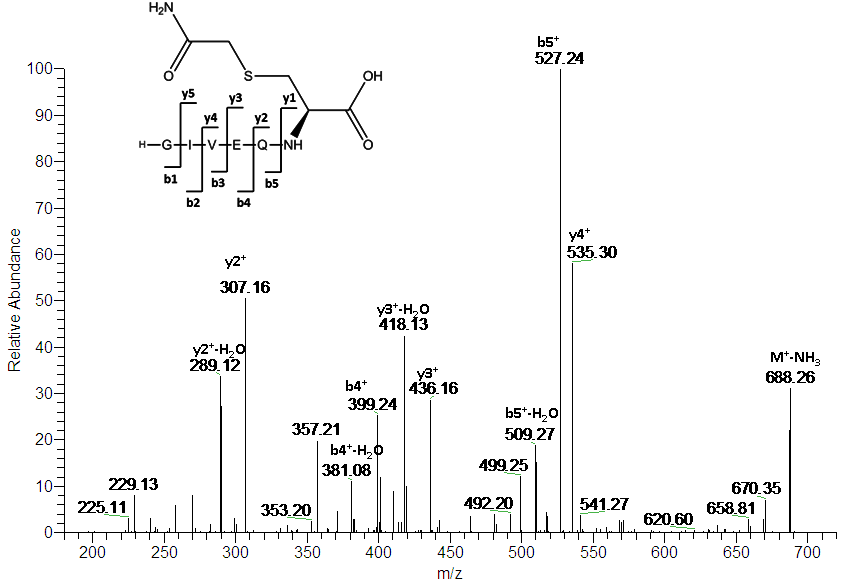


Figure S23


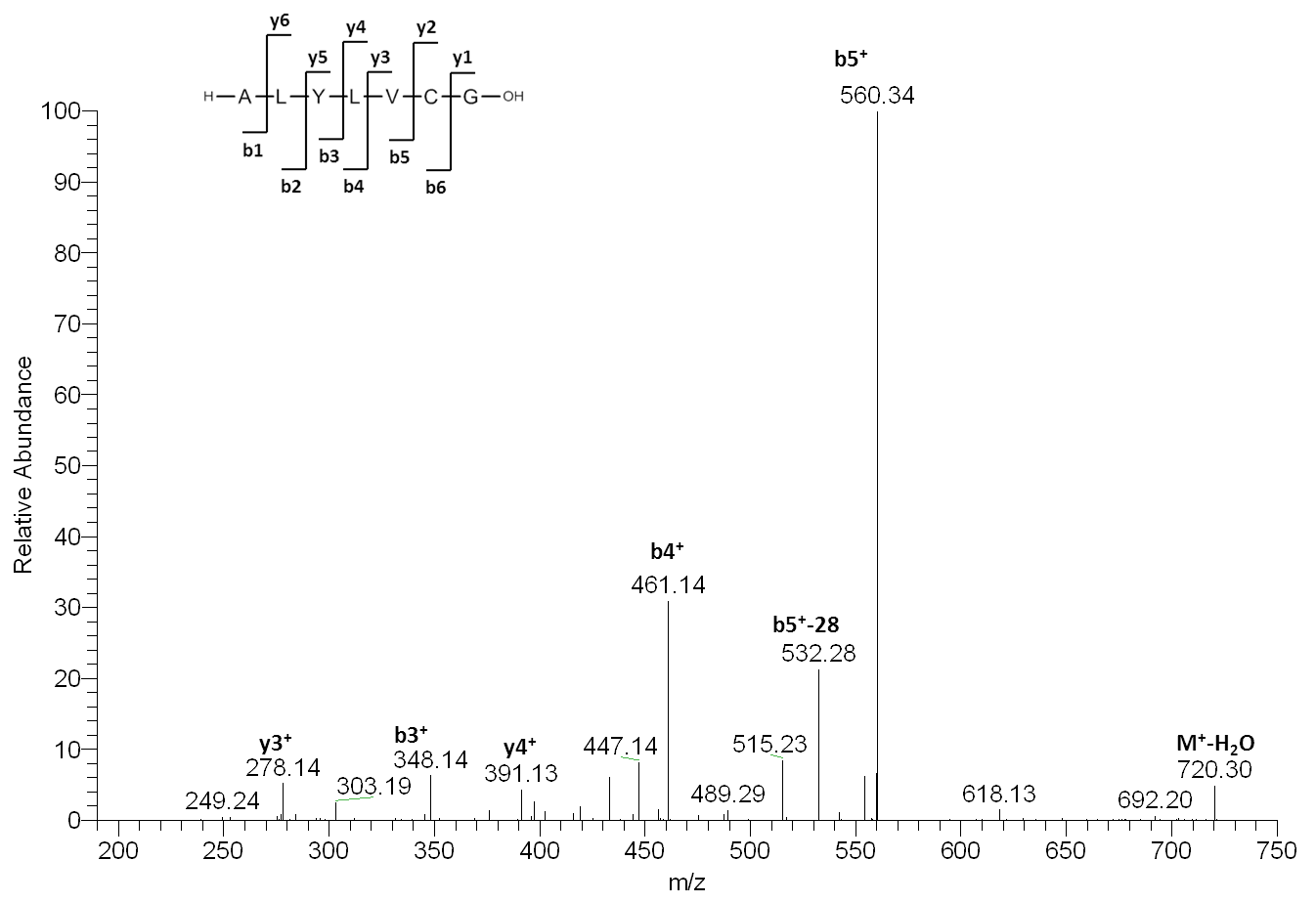


Figure S24

Figure S25

Figure 2

Figure S26


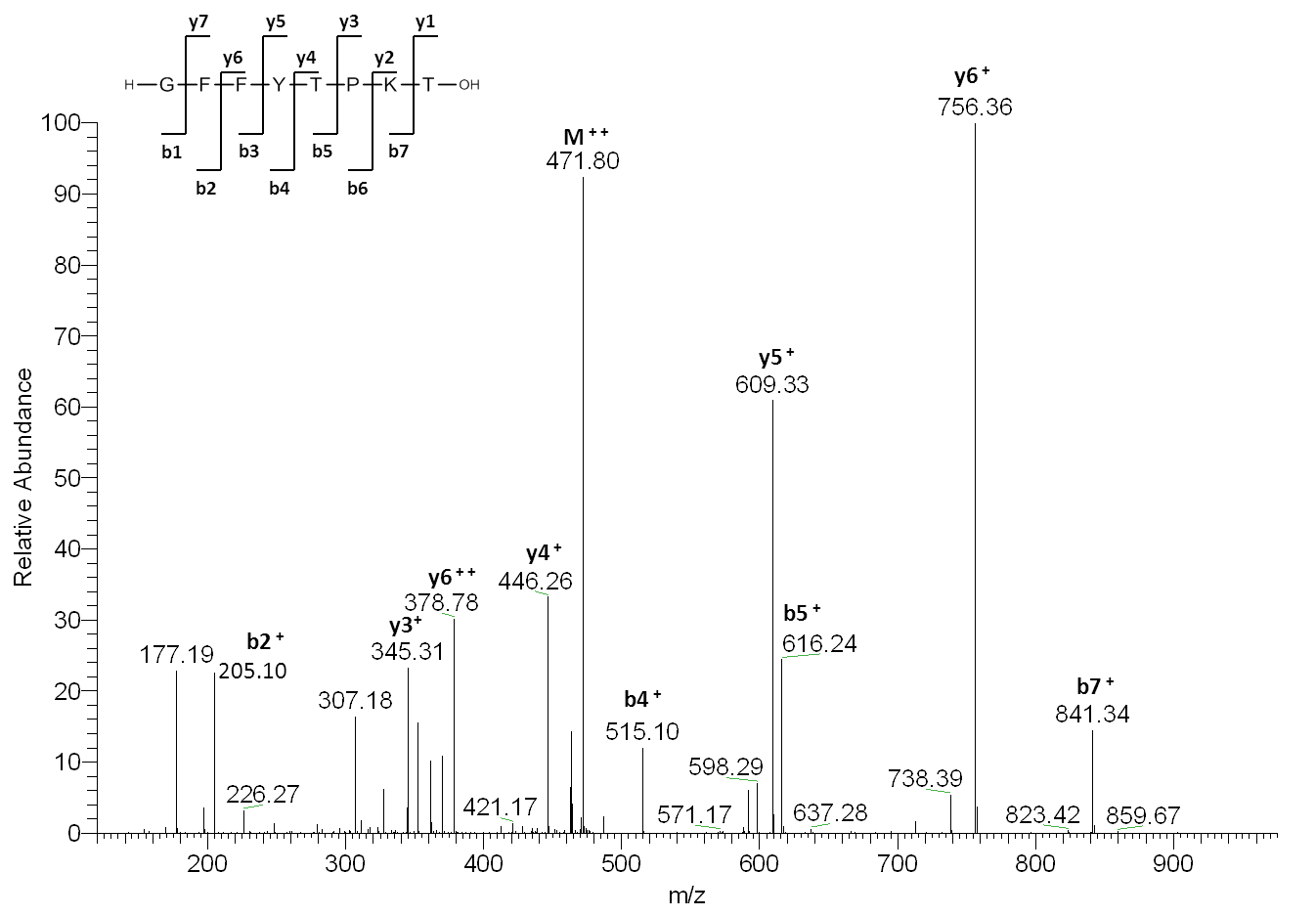


Figure S27

Figure S1 (A, B, C) MS/MS spectra representing coexistence of the oxidized Glu-C fragments R_B22_GFF*YTPKT_B30_ and R_B22_GFFY*TPKT_B30_ measured in oxidized insulin and in the control (native insulin), after reduction (DTT), alkylation (IAM), ABS-derivatization and digestion (Glu-C).

Figure S2  MS/MS spectra representing the oxidized Glu-C fragment A_B14_LY*LVCGE_B21_  measured in oxidized insulin, after reduction (DTT), alkylation (IAM), ABS-derivatization and digestion (Glu-C).

Figure S3   MS/MS spectra representing the oxidized Glu-C fragment F*_B1_VNQHLCGSHLVE_B13_  measured in oxidized insulin, after reduction (DTT), alkylation (IAM), ABS-derivatization and digestion (Glu-C).

Figure S4   MS/MS spectra representing the oxidized Glu-C fragment F**_B1_VNQHLCGSHLVEALYLVCGE_B21_ measured in oxidized insulin, after reduction (DTT), alkylation (IAM), ABS-derivatization and digestion (Glu-C).

Figure S5   MS/MS spectra representing the ABS-derivatized Glu-C fragment R_B22_GFFY^#^TPKT_B30_ measured in oxidized insulin, after reduction (DTT), alkylation (IAM), ABS-derivatization and digestion (Glu-C).

Figure S6   MS/MS spectra representing the ABS-derivatized Glu-C fragment R_B22_GFFY^##^TPKT_B30_  measured in oxidized insulin, after reduction (DTT), alkylation (IAM), ABS-derivatization and digestion (Glu-C).

Figure S7 MS/MS spectra representing the oxidized fragment F*_B1_VNQHLCG_B8_ measured in oxidized insulin, after reduction (DTT), alkylation (IAM), ABS-derivatization and digestion (Glu-C).

Figure S8   MS/MS spectra representing the oxidized fragment E_B21_RGF**FYTPKT_B30_ measured in oxidized insulin, after reduction (DTT), alkylation (IAM), ABS-derivatization and digestion (Glu-C).

Figure S9   MS/MS spectra representing the oxidized fragment F*_B1_VNQHLCGSHLVEAL_B15_ measured in oxidized insulin, after reduction (DTT), alkylation (IAM), ABS-derivatization and digestion (Glu-C).

Figure S10  MS/MS spectra representing the ABS-derivatized fragment A_B14_LY^#^LVC_B19_ measured in oxidized insulin, after reduction (DTT), alkylation (IAM), ABS-derivatization and digestion (Glu-C).

Figure S11    MS/MS spectra representing the ABS-derivatized fragment S_A12_LY^#^QLE_A17_ measured in oxidized insulin, after reduction (DTT), alkylation (IAM), ABS-derivatization and digestion (Glu-C).

Figure S12    MS/MS spectra representing the ABS-derivatized fragment S_A12_LY^##^QLE_A17_ measured in oxidized insulin, after reduction (DTT), alkylation (IAM), ABS-derivatization and digestion (Glu-C).

Figure S13  MS/MS spectra representing the (non-oxidized) fragment L_A13_YQLE_A17_ measured in oxidized insulin and in the control (native insulin), after reduction (DTT), alkylation (IAM), ABS-derivatization and digestion (Glu-C).

Figure S14  MS/MS spectra representing the (non-oxidized) fragment I_A10_CSLY_A14_ measured in oxidized insulin, after reduction (DTT), alkylation (IAM), ABS-derivatization and digestion (Glu-C).

Figure S15  MS/MS spectra representing the (non-oxidized) fragment S_A12_LYQLE_A17_ measured in oxidized insulin, after reduction (DTT), alkylation (IAM), ABS-derivatization and digestion (Glu-C).

Figure S16 MS/MS spectra representing the (non-oxidized) fragment S_A12_LYQL_A16_ measured in oxidized insulin, after reduction (DTT), alkylation (IAM), ABS-derivatization and digestion (Glu-C).

Figure S17 MS/MS spectra representing the (non-oxidized) fragment Y_A14_QLE_A17_ measured in oxidized insulin, after reduction (DTT), alkylation (IAM), ABS-derivatization and digestion (Glu-C).

Figure S18 MS/MS spectra representing the (non-oxidized) fragment G_B8_SHLVE_B13_ measured in oxidized insulin, after reduction (DTT), alkylation (IAM), ABS-derivatization and digestion (Glu-C).

Figure S19 MS/MS spectra representing the (non-oxidized) fragment H_B10_LVEAL_B15_ measured in oxidized insulin, after reduction (DTT), alkylation (IAM), ABS-derivatization and digestion (Glu-C).

Figure S20 MS/MS spectra representing the (non-oxidized) fragment F_B1_VNQHLCGS_B9_ measured in oxidized insulin, after reduction (DTT), alkylation (IAM), ABS-derivatization and digestion (Glu-C).

Figure S21 MS/MS spectra representing the (non-oxidized) fragment Y_B16_LVC_B19_ measured in oxidized insulin, after reduction (DTT), alkylation (IAM), ABS-derivatization and digestion (Glu-C).

Figure S22 MS/MS spectra representing the (non-oxidized) fragment G_B20_ERGFFY_B26_ measured in oxidized insulin, after reduction (DTT), alkylation (IAM), ABS-derivatization and digestion (Glu-C).

Figure S23 MS/MS spectra representing the (non-oxidized) fragment G_A1_IVEQC_A6_ measured in oxidized insulin, after reduction (DTT), alkylation (IAM), ABS-derivatization and digestion (Glu-C).

Figure S24 MS/MS spectra representing the (non-oxidized) fragment A_B14_LYLVCG_B20_ measured in oxidized insulin, after reduction (DTT), alkylation (IAM), ABS-derivatization and digestion (Glu-C).

Figure S25 MS/MS spectra representing the (non-oxidized) fragment Q_A15_LENYCN_A21_ measured in oxidized insulin, after reduction (DTT), alkylation (IAM), ABS-derivatization and digestion (Glu-C).

Figure S26 MS/MS spectra representing the (non-oxidized) fragment L_B6_CGSHLVE_B13_ measured in oxidized insulin, after reduction (DTT), alkylation (IAM), ABS-derivatization and digestion (Glu-C).

Figure S27 MS/MS spectra representing the (non-oxidized) fragment G_B23_FFYTPKT_B30_ measured in native insulin, after reduction (DTT), alkylation (IAM), ABS-derivatization and digestion (Glu-C).
